# Identification, characterization and expression profiles of E2 and E3 gene superfamilies during the development of tetrasporophytes in *Gracilariopsis lemaneiformis* (Rhodophyta)

**DOI:** 10.1186/s12864-023-09639-0

**Published:** 2023-09-18

**Authors:** Qiong Wu, Jingru Yin, Min Jiang, Jingyu Zhang, Zhenghong Sui

**Affiliations:** Key Laboratory of Marine Genetics and Breeding, Ministry of Education, Ocean University of China), Qingdao, 266003 China

**Keywords:** *Gracilariopsis lemaneiformis*, E2 ubiquitin conjugating enzymes, E3 ubiquitin ligases, Tetrasporophytes development and tetraspores release

## Abstract

E2 ubiquitin conjugating enzymes and E3 ubiquitin ligases play important roles in the growth and development of plants and animals. To date, the systematic analysis of E2 and E3 genes in Rhodophyta is limited. In this study, 14 E2 genes and 51 E3 genes were identified in *Gracilariopsis lemaneiformis*, an economically important red alga. E2 genes were classified into four classes according to the structure of the conserved domain, UBC. E3 genes were classified into 12 subfamilies according to individual conserved domains. A phylogenetic tree of seven algae species showed that functional differentiation of RING-type E3s was the highest, and the similarity between orthologous genes was high except in *Chlamydomonas reinhardtii* and *Chara braunii*. RNA-seq data analysis showed significant differential expression levels of E2 and E3 genes under the life stages of tetraspore formation and release, especially *GlUBCN* and *GlAPC3*. According to GO and KEGG analysis of two transcriptomes, GlUBCN and GlAPC3 were involved in ubiquitin-mediated proteolysis, and other subunits of the anaphase promoting complex or cyclosome (APC/C) and its activators GlCDC20 and GlCDH1 were also enriched into this process. The *CDH1* and *CDC20* in 981 were down-regulated during tetraspores formation and release, with the down-regulation of *CDH1* being particularly significant; *CDH1* and *CDC20* in WLP-1, ZC, and WT were up-regulated during tetraspores formation and release, with *CDC20* being more significantly up-regulated. Therefore, *GlCDH1*, rather than *GlCDC20*, in ‘981’ might play the leading role in the activation of the APC/C, and *GlCDC20* might play the leading role rather than *GlCDH1* in strains WLP-1, ZC and wild type. The low fertility of cultivar 981 might be highly correlated with the inactivity of activators CDH1 and CDC20. This study provided a basic and comprehensive understanding of characteristic of E2 and E3 genes in *Gp. lemaneiformis* and set a foundation for further understanding of E2 ubiquitin conjugating enzymes and E3 ubiquitin ligase in regulating tetrasporophytes development of *Gp. lemaneiformis*.

**Supplementary Information:**

The online version contains supplementary material available at 10.1186/s12864-023-09639-0.

## Background

Ubiquitination, which is a covalent binding process between ubiquitin and target proteins in the ubiquitin proteasome system (UPS), is one kind of post-translational modification of proteins [1]. The UPS consists of ubiquitin, E1 ubiquitin activating enzyme (E1), E2 ubiquitin conjugating enzyme (E2), E3 ubiquitin ligase (E3) and 26S proteasomes [2]. Firstly, the C-terminal glycine carboxyl of a ubiquitin chain is linked to the cysteine active site of an E1 in an ATP-dependent manner. Subsequently, the activated ubiquitin chain is transferred to the cysteine active site of an E2 by trans-esterification with the E1 [3], and an E3 ubiquitin ligase mediates the directional transfer of the ubiquitin to the substrate protein [4]. Finally, the substrate proteins are recognized and degraded by a 26S proteasome complex in an ATP-dependent manner, and the ubiquitin chain is hydrolyzed to single ubiquitin molecules [5, 6].

Compared with E1s and E2s, E3 ubiquitin ligase recognizes specific substrates [7]. In eukaryotes, there are no more than two E1s and a few dozen E2s, but there are hundreds of E3s [8]. In *Arabidopsis thaliana*, for example, there are more than 1300 E3s in the genome, but only two types of E1 and 37 types of E2 [9]. In humans, there are more than 600 E3s, but only two E1s and 40 E2s [10]. E3s recruit many different substrates by directly mediating the binding of ubiquitin to substrate proteins. In this way, the 26S proteasome can hydrolyze numerous substrate proteins in organisms, making the UPS multifunctional [11]. Therefore, E3 ubiquitin ligase is recognized as the most diverse and important enzyme in the ubiquitination pathway.

According to their conserved domains and mechanisms of mediating the combination of ubiquitin and substrate proteins, E3 ligases have been divided into three types: RING (Really Interesting New Gene), HECT (Homologous to E6-APC Terminus) and RBR (RING Between RING) type [12]. RING E3s are the most abundant, comprising RING [13], U-box [14], Cullin-RING [15, 16] and the anaphase promoting complex/cyclosome (APC/C) [17, 18], although they do not necessarily form one superfamily. The Zn-binding domain of RING type E3s recruits Ub-charged E2, and the U-box type is in the same fold as RING, only without Zn coordination [19, 20]. Monomeric RING, homodimeric RING and heterodimeric RING E3s belong to the RING type, while monomeric U-box and homodimeric U-box E3s belong to the U-box type [21, 22]. Cullin-RING and APC/C types are E3 ubiquitin ligase complexes: Cullin-RING complexes are composed of a substrate receptor (SR), adaptor protein, cullin and ring-box protein (Rbx) [23, 24]. The APC/C can be divided into at least 11 subunits, depending on the species [25]. In humans, for example, the APC/C is a large assembly of 19 subunits, which includes both a RING (APC11) and Cullin-RING (APC2) [26]. The E3 ligases of the HECT domain type can be classified into three families based on their N-terminal differences: the Nedd4 family, HERC (HECT and RCC1-like domain) family, and other HECTs [27]. In these E3s, at least one HECT domain is present in addition to several WW (tryptophan-tryptophan) motifs in Nedd4 family members, and at least one RLD (RCC1-like domains, RCC1: regulator of chromosome condensation 1) is contained in HERC family members at the C-terminus [28]. RBR E3s have been reported in recent years. They are similar to the RING type in structure and the HECT type in their mechanism [29]. Two RING domains (RING1 and RING2) are separated by an in-between-RING domain (IBR, also called BRcat). RING1 recruits the E2, and RING2 possesses the catalytic cysteine. Because RING2 is not a standard structure of the RING type domain, it is also called Rcat (required-for-catalysis). The structure of the IBR domain is the same as the RING2 domain, but lacks the catalytic cysteine [30].

Ubiquitination is an extremely sensitive, rapid and reversible regulation process and involves complex modes of protein modification with diverse results [3]. In plants, the UPS and ubiquitination play crucial roles in various life processes, such as circadian rhythm [31], flowering time [32], cell cycle [33], abiotic stress [34], phytohormones signal response [35–39], DNA damage repair [40], plant immunity [41], and so on [42–49].

**Fig. 1:**
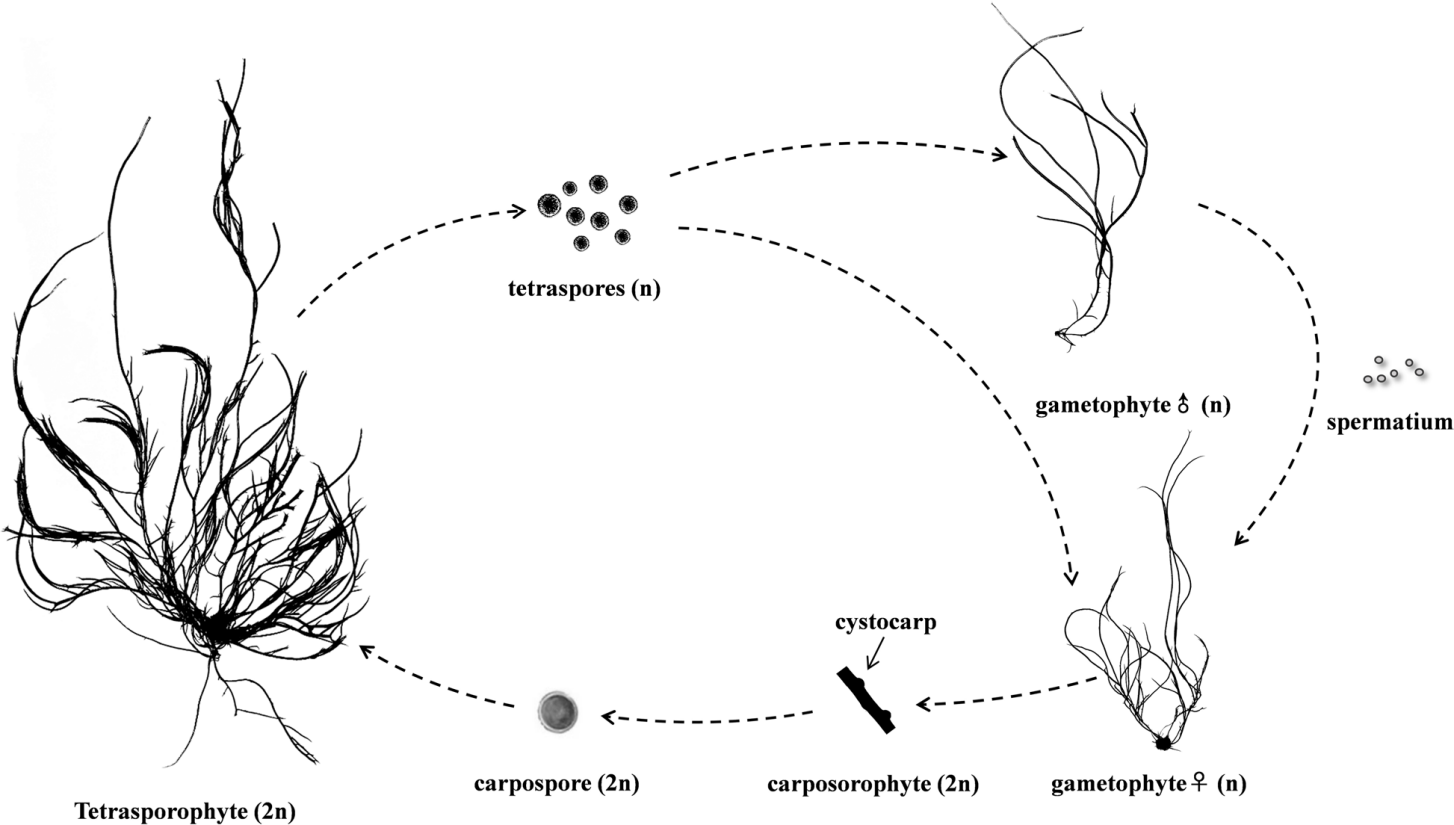
The life story of *Gp. lemaneiformis*

*Gracilariopsis lemaneiformis* is an economically important red macroalgae [50–53], the life history of which is characterized by alternation of generations [54]. In the tetrasporophyte generation, haploid tetraspores are produced by tetrasporophytes. During tetraspores formation, tetrasporangium cells are formed between the epidermal cells, and the cells develop to maturation after meiosis. Then, they are carried away from the organism, and tetraspores are released. From this point, a period of tetraspores release begins [55, 56]. The life history of *Gp. lemaneiformis* was shown as follows (Fig. 1).

Our previous study indicated that the expression level of some E3 genes, including *CDC53* (Cullin-RING type), *APC3* (CDC27, APC/C type), *COP1* (heterodimeric RING type), and *SDIR1* (RING type), varied during tetraspores formation and release, and it was suggested that these E3 genes might regulate DNA duplication and chromosome morphological changes [57]. Therefore, it was supposed that E3 ubiquitin ligases might play a regulatory role during tetraspores formation and release.

To date, no systematic analysis of E2 and E3 genes in Rhodophytes has been reported. The aim of this study was to systematically analyze the ubiquitin system of *Gp. lemaneiformis*, as well as to provide directional evidence of the associate relationship of E2 and E3 genes to the tetrasporophyte development. The study will further our understanding of E3 ubiquitin ligases and their role in regulating tetrasporophyte development, while also providing a theoretical basis for developing strains with economical traits in the future.

## Results

### Identification of E2 and E3 genes in Gp. lemaneiformis

The whole genome database of *Gp. lemaneiformis* (SRR20338037) was searched. Genes associated with the UPS were identified, including ubiquitin, E1 ubiquitin binding enzymes, E2 ubiquitin activating enzymes (also called GlUBCs), and E3 ubiquitin ligases. In this study, we focused on E2 ubiquitin activating enzymes and E3 ubiquitin ligases, identifying 14 and 51 of each, respectively.

**Fig. 2:**
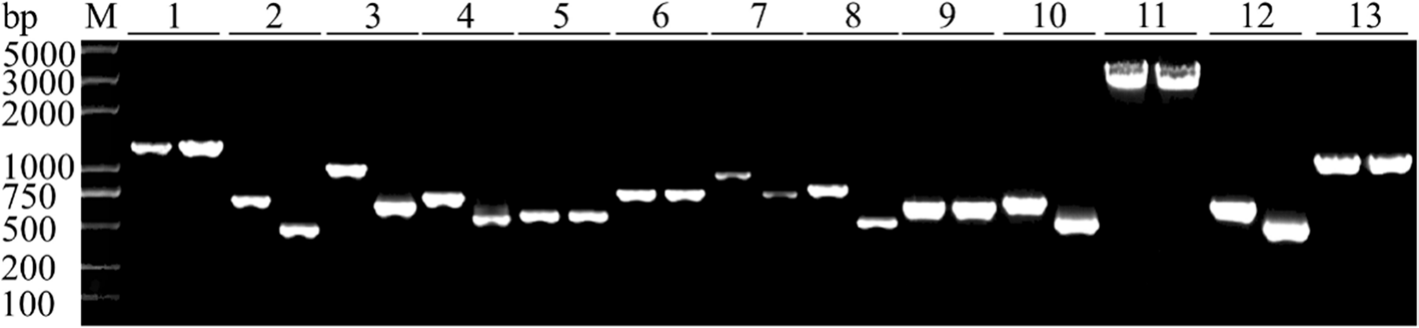
PCR amplification products of 13 E2 genes of wild type of *Gp. lemaneiformis*. M: marker. Number 1–13 indicated different E2 ubiquitin activating enzyme genes, and the left were DNA sequences, and the right were cDNA sequences

**Fig. 3:**
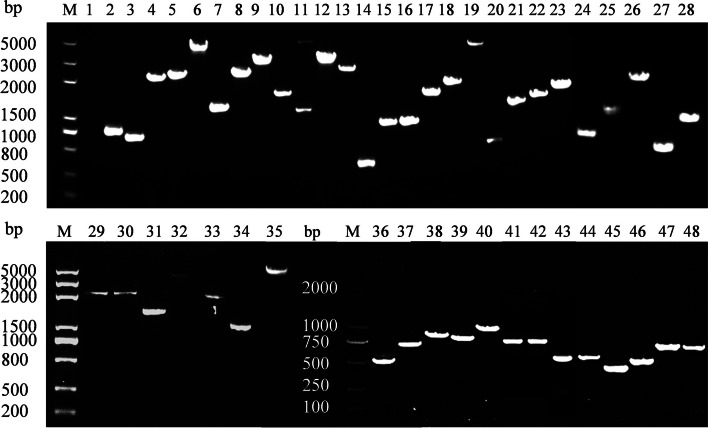
PCR amplification products of 48 E3 genes’ cDNA of wild type of *Gp. lemaneiformis*. M: marker. Number 1–48 indicated cDNA sequences of different E3 ubiquitin ligase genes

First, all E2 ubiquitin activating enzyme genes and 48 E3 ubiquitin ligase genes with a CDS sequence base number less than 6,000 bp were amplified to verify whether these genes could be transcribed. All E2 genes (Fig. 2, Fig. S1) and 48 E3 genes (Fig. 3, Fig. S2) could be transcribed, while three E3s larger than 6,000 bp were too long to be amplified. In Fig. 2, some E2 genes displayed length differences between the DNA and transcript, suggesting that these genes contained intron(s).

Some basic physicochemical properties, such as molecular weight, instability index, isoelectric point (pI) and grand average of hydropathicity (GRAVY) were analyzed.

**Fig. 4:**
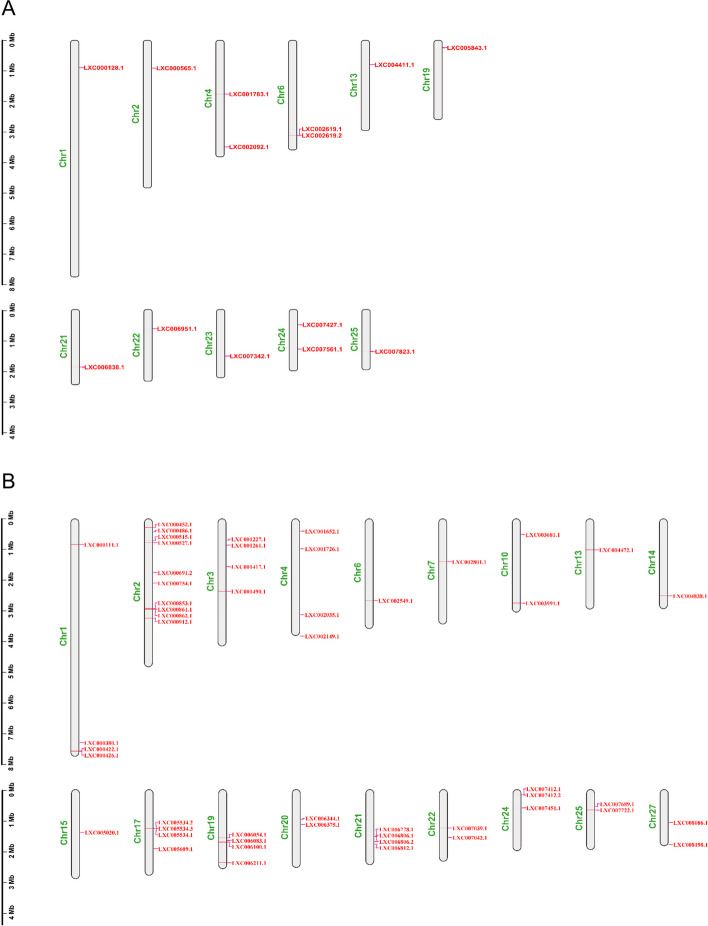
E2 (**A**) and E3 (**B**) gene localization on chromosomes of *Gp. lemaneiformis*. The scale bar beside the chromosome indicated the length in megabases (Mb)

For E2s (Supplementary Table S1), the number of exons ranged from 1 to 5, and the molecular weights of the proteins ranged from 16.2 to 96.5 kDa (142 to 879 amino acids). The E2 genes were evenly located on 10 chromosomes (Chr1, Chr4, Chr6, Chr13, Chr19, Chr21, Chr22, Chr23, Chr24, and Chr25) (Supplementary Table S1, Fig. 4A), and the instability indexes ranged from 38.54 to 68.57, which indicated that all of the E2s were unstable proteins (instability index > 40) except GlUBC1. The pI values ranged from 4.32 (GlUBC5) to 10.01 (GlUBCJ2), and the results of GRAVY showed that all the E2 genes encoded hydrophilic proteins.

For E3s (Supplementary Table S2), the range of exons was the same as that of E2 genes, and the molecular weights of the proteins ranged widely from 12.5 to 574.3 kDa (109 to 5172 amino acids). The E3 genes were located on 18 chromosomes (Chr1, Chr2, Chr3, Chr4, Chr6, Chr7, Chr10, Chr13, Chr14, Chr15, Chr17, Chr19, Chr20, Chr21, Chr22, Chr24, Chr25, and Chr27) (Supplementary Table S2, Fig. 4B). The instability index values ranged from 21.64 to 83.69, and hence, only eight E3 genes were stable proteins. The pI values ranged from 4.62 (GlSUD1) to 10.36 (GlPQT3), and the GRAVY results showed that almost all E3 genes encoded hydrophilic proteins, except GlRNF12-2 (0.204), GlSUD1 (0.048), GlHIP1 (0.02), and GlTME3 (0.176).

### Chromosome localization of E2 and E3 gene members

Fourteen E2 genes were distributed on 11 of the 28 chromosomes (Fig. 4A). There were two E2 genes located on Chr4, Chr6, and Chr24, respectively. Chr1, Chr2, Chr13, Chr19, Chr21, Chr22, Chr23, Chr24, and Chr25 contained only one E2 gene each. There were two copies of only one E2 gene, *GlUBC5* (LXC002619.1, LXC002619.2).

For E3s, 51 genes were mapped more widely, on 18 chromosomes (Fig. 4B). The number of genes on each chromosome was irrelevant to chromosome size. The highest density of E3 genes occurred on Chr2, 10 members, while the largest chromosome (Chr1) carried only four E3 genes. Chr6, Chr7, Chr13, Chr14, and Chr15 contained one E3 gene each, while the smallest chromosome (Chr27) contained two E3 genes. The maximum and minimum numbers of genes were respectively mapped to Chr19 and Chr3. There were two copies of *GlWWP1* and *GlMIB2*, and three copies of *GlsconC*, and the remaining E3 genes were single copy.

### Phylogenetic evolution, gene structure and conserved motif analysis

To explore the classification and evolutionary relationships of E2 and E3 members, phylogenetic trees were constructed, and gene structures were visualized, while motif compositions were also investigated.

**Fig. 5:**
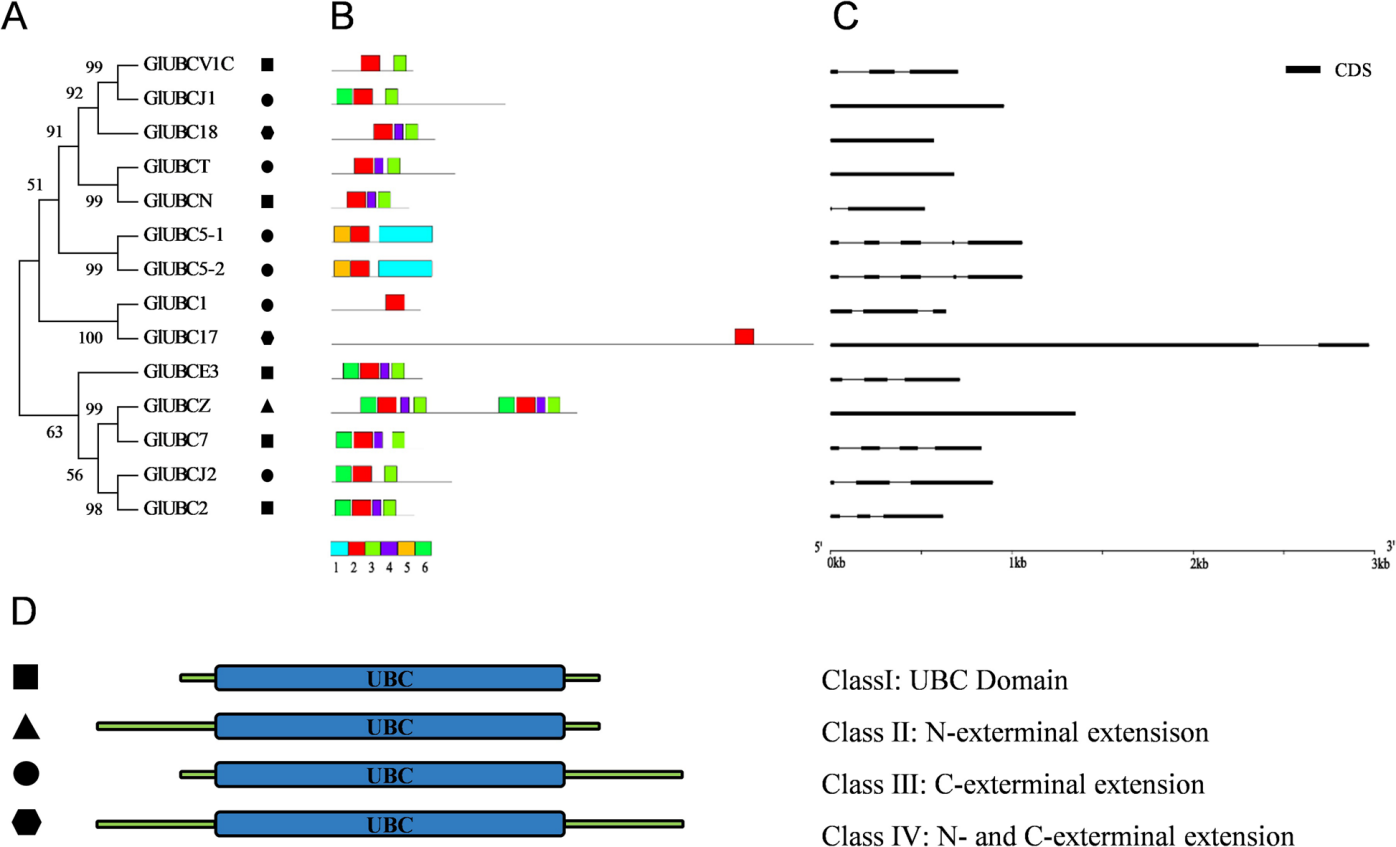
The phylogenetic tree, gene structures and protein motifs of *Gp. lemaneiformis* E2 ubiquitin conjugating enzymes. **A** The phylogenetic tree. **B** Protein motifs. The colorful boxes delineated different motifs. **C** Gene structures. Exons were displayed using black bars. Black lines denoted introns. **D** Classification of E2 ubiquitin conjugating enzymes. The proteins were divided into four classes: class I, class II, class III, class IV, and represented by different shapes. The tree was constructed by MEGA7 using ML with 1000 bootraps

In E2 proteins, there was only one conserved domain, named UBC (smart00212, pfam00179) (Supplementary Fig. S3). Six conserved motifs of E2 proteins were found (Fig. 5B), and the most highly conserved was motif 2, which occurred in each E2 protein. The E2 genes could be divided into two groups according to the phylogenetic trees (Fig. 5A), and their sizes varied. The smaller one seemed to be more conserved, because the conserved motifs were arranged in a similar way. The results of the structure analysis (Fig. 5C) showed that there was little correlation between the conserved motifs and gene structures. For example, the motifs of GlUBC2 and GlUBCZ were the same, but their structures differed. Based on the conserved domain analysis and previous research on higher plants [58], the E2 proteins in *Gp. lemaneiformis* were divided into four categories (Fig. 5D): class I (5 members), class II (1 member), class III (6 members) and class IV (2 members), which were defined as containing only a single UBC domain, a plus N-exterminal extension, a plus C-exterminal extension, or both N-exterminal and C-exterminal extensions, respectively. These extensions were associated with functional differences between E2 genes, such as the stability of the interaction with E1 ubiquitin-activating enzymes and the activity of interaction with E3 ubiquitin ligases [59].

**Fig. 6:**
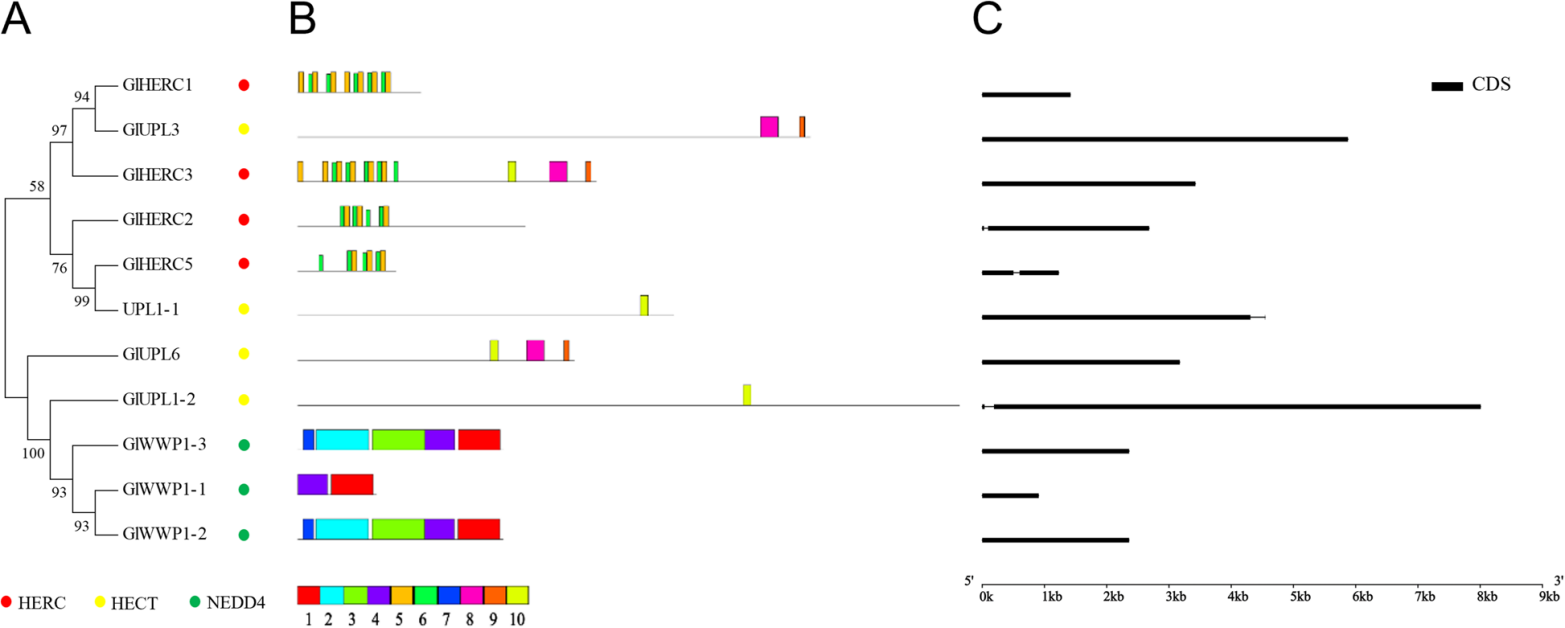
The phylogenetic tree, protein motifs and gene structures of HECT-type E3 ubiquitin ligases in *Gp. lemaneiformis*. **A** The phylogenetic tree. **B** Protein motifs. The colorful boxes delineated different motifs. **C** Gene structures. Exons were displayed using black bars. Black lines denoted introns

**Fig. 7:**
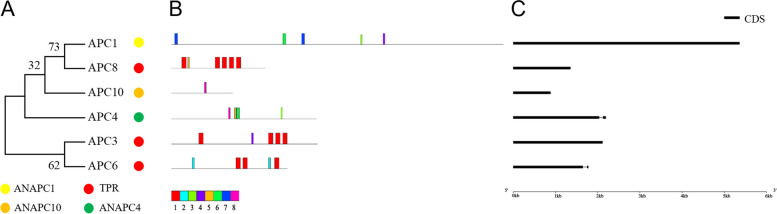
The phylogenetic tree, protein motifs and gene structures of APC/C-type E3 ubiquitin ligases in *Gp. lemaneiformis*. **A** The phylogenetic tree. **B** Protein motifs. The colorful boxes delineated different motifs. **C** Gene structures. Exons were displayed using black bars. Black lines denoted introns

**Fig. 8:**
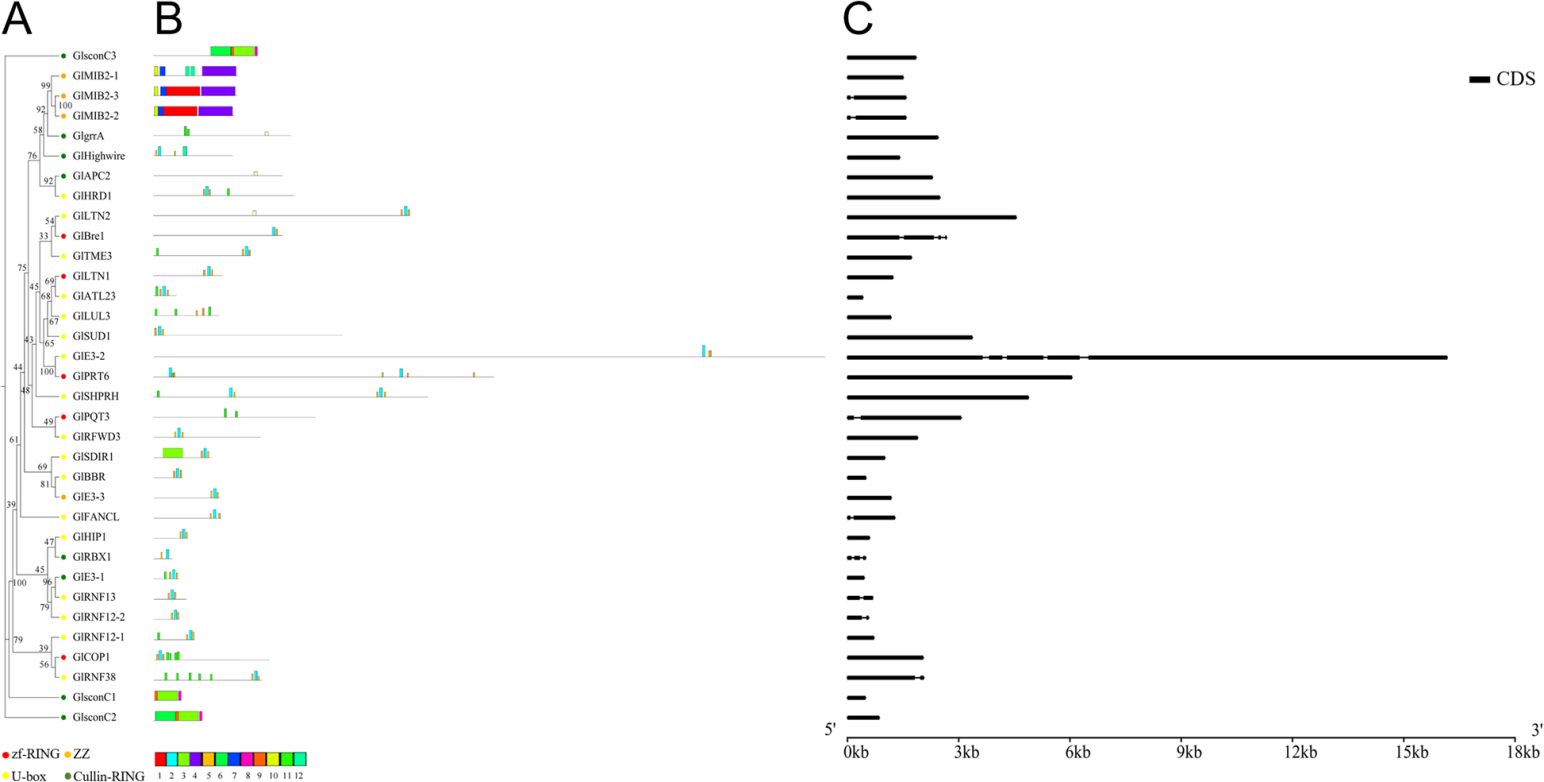
The phylogenetic tree, protein motifs and gene structures of RING-type E3 ubiquitin ligases in *Gp. lemaneiformis*. **A** The phylogenetic tree. **B** Protein motifs. The colorful boxes delineated different motifs. **C** Gene structures. Exons were displayed using black bars. Black lines denoted introns

E3 genes in *Gp. lemaneiformis* were divided into the HECT family, APC/C family and RING superfamily (Figs. 6, 7 and 8). The HECT family was subdivided to NEDD4 (including WW conserved domain), HERC (including HERC conserved domain), and HECT (including HECT conserved domain) types, respectively, containing 3, 4, and 4 E3 genes, respectively (Fig. 6A, Fig. S4). The APC/C contained all subunits of the APC complex except APC2 (of the Cullin-RING type) and the conserved domain TPR (tetratricopeptide repeats), the presence of which suggested protein–protein interaction [60, 61] occurred in APC3, APC6, and APC8 (Fig. 7A, Fig. S5). The RING type was divided into four subclasses, Cullin-RING, U-box, ZZ type, and Zn finger-RING (zf-RING), according to the conserved domains, which contained 8, 17, 4 and 5 E3 genes, respectively (Fig. 8A, Fig. S6).

For the HECT family, the phylogenetic trees analysis (Fig. 6A) showed that members were clustered into two branches, and the HECT-type (yellow dots) showed functional differentiation. The results of the protein motif analyses (Fig. 6B) showed that the classification of motifs was exactly in accordance with the classification of the conserved domains (Fig. S4) but not exactly in accordance with the gene structures (Fig. 6C). There were two common motifs (motifs 5 and 6) in the HERC family, and two common motifs (motifs 1 and 4) in the NEDD4 family. Motif 10 was present in the HECT family, except in UPL3. However, motifs 8 and 9 occurred in UPL3, UPL6, and HERC3.

In the APC/C, six members were divided to two branches according to the phylogenetic tree analysis (Fig. 7A). APC3 and APC6 were clustered to the same branch, and the others were clustered together. The results of protein motif and conserved domain analysis (Fig. 7B, Fig. S5) revealed that both APC3 and APC6 contained several repeats of ‘motif 1’ and conserved domain ‘ANAPC3’, as well as ‘TPR repeats’. There was one common motif (motif 8) in APC4 and APC10, and one common motif (motif 6) in APC4 and APC1. The motifs existed in specific groups, which might be related to their specific biological functions. It should be noted that APC2 was of the Cullin-RING type, but not the APC/C type of E3 ubiquitin ligases.

In the RING-type, members were divided into four groups according to the phylogenetic trees (Fig. 8A). sconC1, sconC2 and sconC3 were distributed on different branches separately, and the other members were clustered into a large branch. The results of the protein motif analysis (Fig. 8B) revealed that all sconC proteins contained the structure ‘motif 9- motif 3- motif 8’ and all MIB2 proteins contained the structure ‘motif 10- motif 7- motif 4’. GrrA, LUL3, and PQT3 contained two or three repeats of ‘motif 11’, however they were not clustered together. ‘Motif 10’ was contained in APC2 and was also present in GrrA and LTN2. The rest of the E3 ubiquitin ligases contained the structure ‘motif 5-motif 2-motif 5’ or ‘motif 2-motif 5’, and ‘motif 11’ was also included in many of them. According to the above results, the types of motifs and evolutionary relationships were not exactly in accordance with each other.

The gene structure analysis (Figs. 6C, 7C and 8C) showed that the lengths of E3 genes varied significantly. The longest E3 gene was *GlE3-2* (16.17 kb) and the shortest was *GlATL23* (0.42 kb), both of which were U-box type E3 genes (Fig. 8C). It was also found that there was no specific relationship between gene length and E3 type, and between gene length and number of introns. This huge difference in their form and structure indicated that they were likely to participate in various processes during *Gp. lemaneiformis* development. In addition, the density of introns in E3 genes was much lower than that in E2 genes of *Gp. lemaneiformis.* Most E2 genes contained multiple introns, while there were no introns in most E3 genes.

**Fig. 9:**
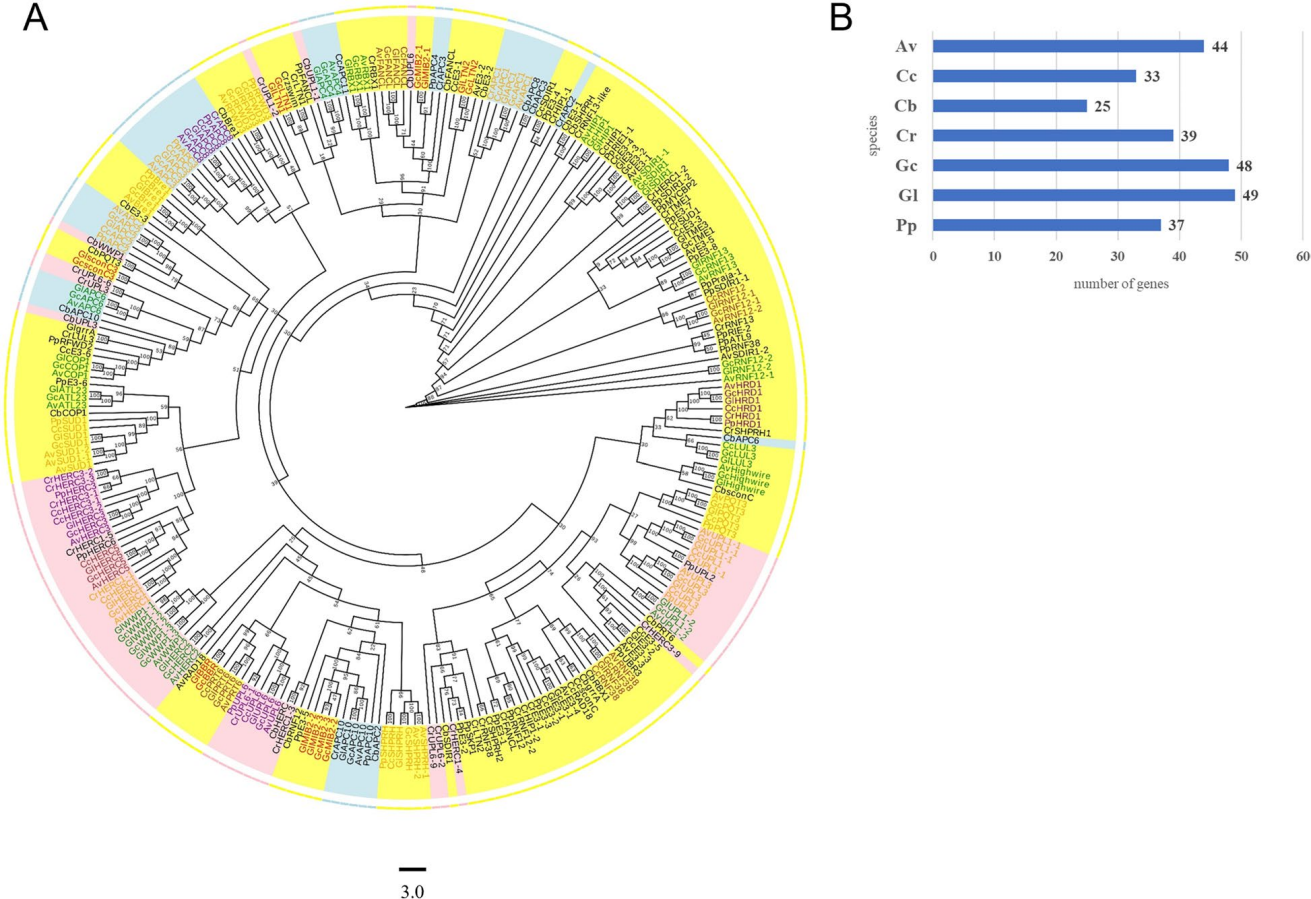
Phylogenetic and evolutionary analysis of E3 genes family in seven species of algae. **A** The phylogenetic tree. **B** The number of E3 genes in the seven algae. Av, *Agarophyton vermiculophylla*, Cb, *Chara braunii,* Cc, *Chondrus crispus*, Cr, *Chlamydomonas reinhardtii*, Gc, *Gracilariopsis chorda*, Gl, *Gracilariopsis lemaneiformis*, Pp, *Porphyridium purpureum.* The background of yellow, pink and blue represented RING-type, HECT-type, and APC/C E3 ubiquitin ligases, respectively. The clustering analysis was based on 1000 replications for increasing the credibility of the bootstrap value

An unrooted phylogenetic tree from seven species was constructed (Fig. 9A), which included 275 proteins from *Agarophyton vermiculophylla*, *Chondrus crispus*, *Chara braunii*, *Chlamydomonas reinhardtii*, *Gp. chorda*, *Gp. lemaneiformis*, and *Porphyridium purpureum*, and the number of orthologous E3 genes in these species were counted (Fig. 9B). As shown in Fig. 9A, most E3 genes of these algae were RING-type genes marked with yellow background, and the degree of differentiation of RING-type E3s was the highest. As for HECT-type genes (marked with pink background), the degree of differentiation of *UPL1-1* and *UPL3* were the highest. The highest degree of differentiation of APC subunits (marked with blue background) was APC10, and there was the highest similarity between APC3 and APC8.

The orthologous genes in *Gp. chorda* and *Gp. lemaneiformis* showed the highest similarity, as all of them were arranged adjacently on the same branch. *Agarophyton vermiculophylla*, *Gp. chorda*, and *Gp. lemaneiformis* orthologous genes, *RBX1*, *APC4*, *LUL3*, *HIP1, SDIR1*, *RNF13*, RNF12-2, *UPL1-2*, *Highwire*, *HERC2*, *WWP1*, *ATL23*, *COP1* and *APC6* (gene names in green), were clustered on the same branch and adjacent. Among them, *HIP1, SDIR1*, *UPL1-2*, *HERC2*, *WWP1* and *APC6* in these three species were on proprietary branches of *Gracilaria*, which suggested that these genes may be related to the functional specialization of *Gracilaria* species. The orthologous genes in *Agarophyton vermiculophylla*, *Chondrus crispus*, *Gp. chorda*, and *Gp. lemaneiformis*, *FANCL*, RNF12-1, *RNF38*, *PRT6* and *HERC5* (gene names in brown), were clustered to the same branch and adjacent. Moreover, the orthologous genes in *Agarophyton vermiculophylla*, *Chondrus crispus*, *Gp. Chorda*, *Gp. lemaneiformis*, and *Porphyridium purpureum/ Chlamydomonas reinhardtii*, *APC1, PQT3*, *UPL1-1*, *UPL3*, *SHPRH*, *HERC1*, *SUD1*, *APC2*, *Bre1*, *APC3* and *RFWD3* (gene names in orange) were clustered on the same branch and adjacent. All *APC8*s, *HERC3*s, *UPL6*s and *HRD1*s in the seven species were separately gathered together (gene name in purple), the differentiation of which were very conserved, and might be of great significance during the growth and development process of these species. The similarity of all E3 genes in *Chara braunii* was low with E3 orthologous genes in other six algae. In a word, according to the position of the orthologous genes on the phylogenetic tree, the order of similarity of E3 ubiquitin ligase genes between *Gp. lemaneiformis* and the other six species, from high to low, was *Gp. chorda*, *Agarophyton vermiculophylla*, *Chondrus crispus*, *Porphyridium purpureum*, *Chlamydomonas reinhardtii* and *Chara braunii*.

### Analysis of cis-acting elements in E3 genes of Gp. lemaneiformis

**Fig. 10:**
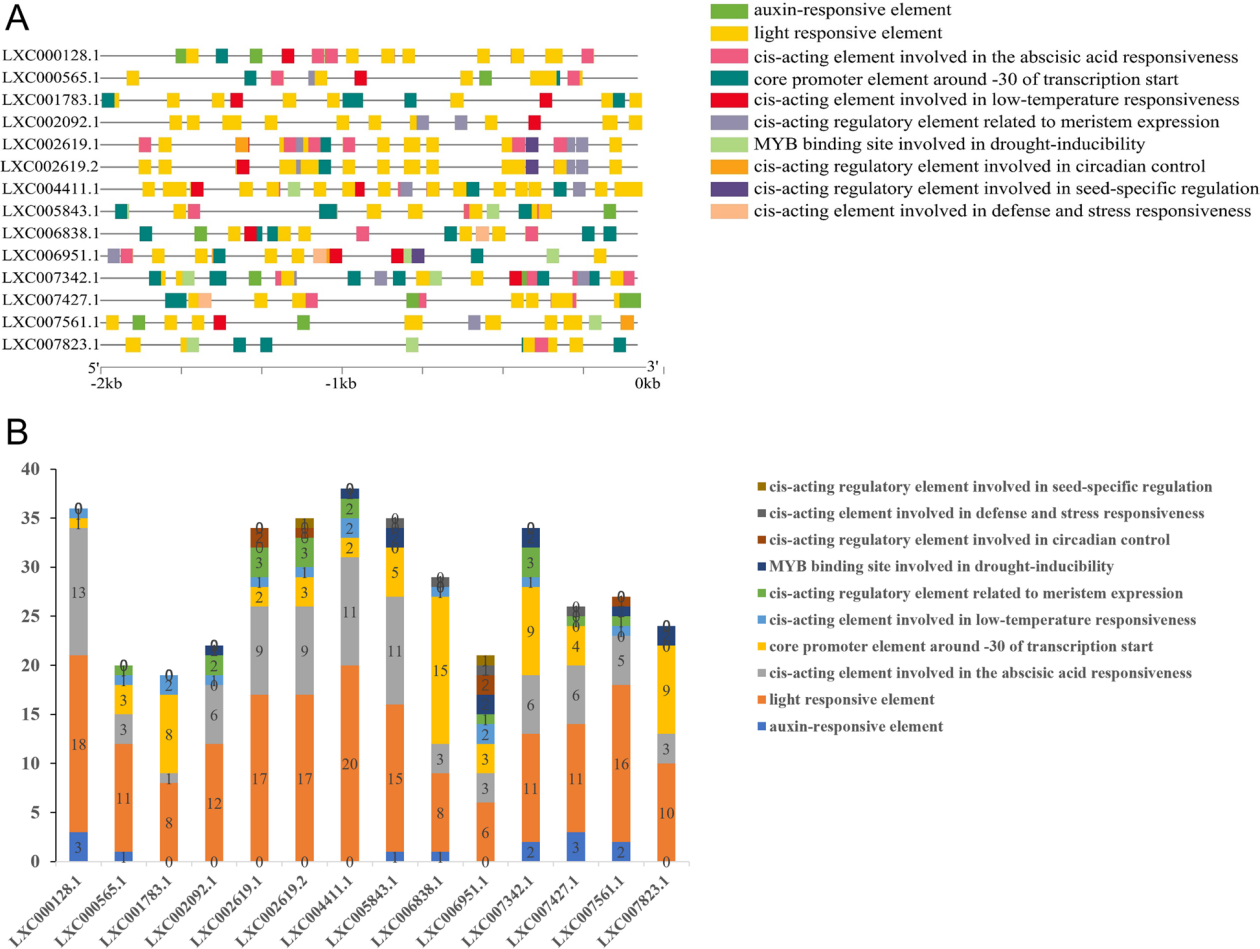
The cis-elements of promoter region (**A**) and quantity statistics (**B**) of E2 genes in *Gp. lemaneiformis*. The 2 kb upstream of coding sequence of 14 E2 genes were predicted by the PlantCARE. The different colored blocks denoted different type of cis-elements

**Fig. 11:**
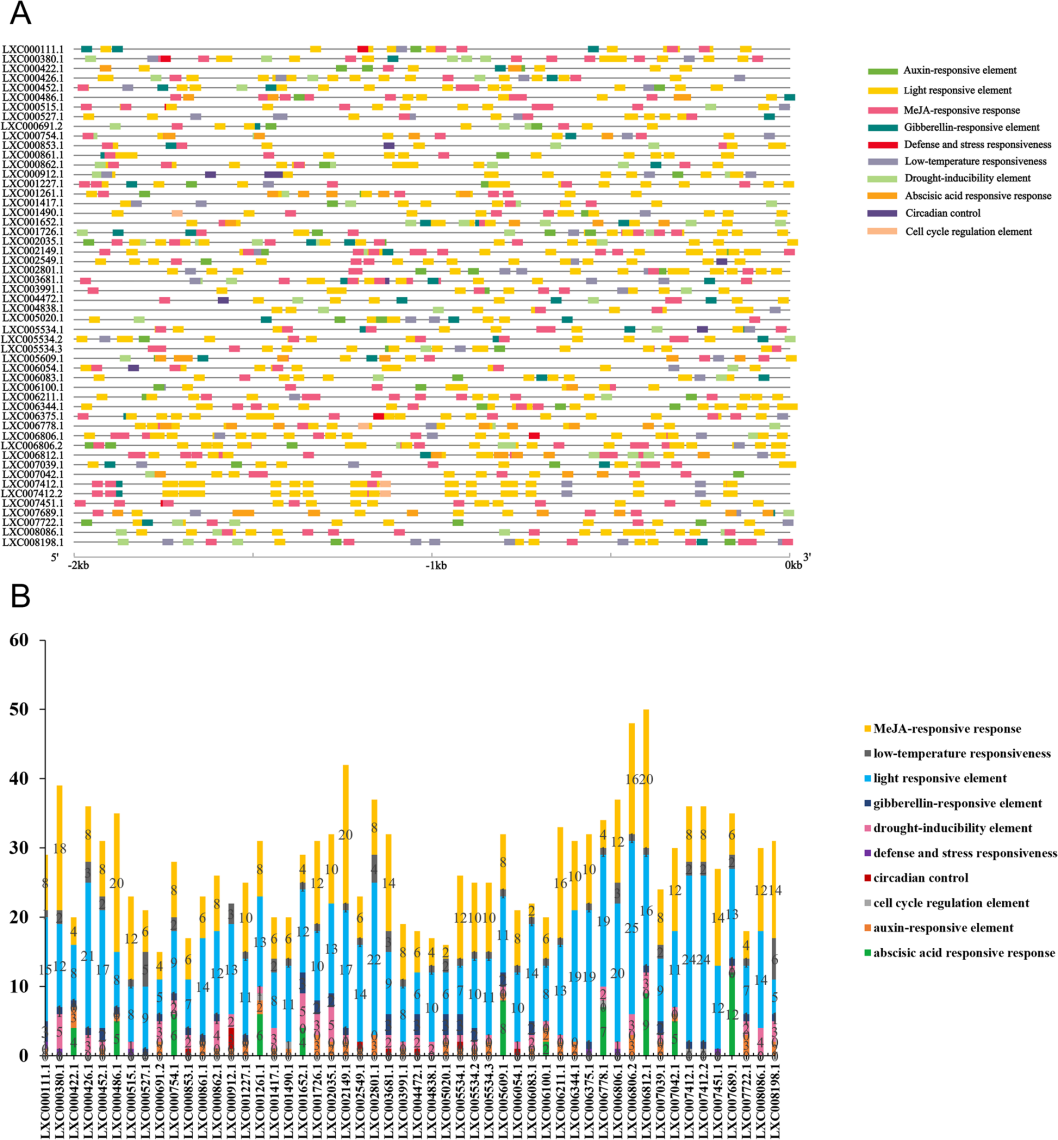
The cis-elements of promoter region (**A**) and quantity statistics (**B**) of E3 genes in *Gp. lemaneiformis*. The 2 kb upstream of coding sequence of 51 E3 genes were predicted by the PlantCARE. The different colored blocks denoted different typical of cis-elements

To better understand the transcriptional regulation mechanism of the upstream promoters of E2 and E3 genes, the sequences of these identified E2 and E3 gene promoter regions (2000 bp region upstream) were submitted to and detected by PlantCARE, and a column chart was established to display the results according to the frequency their appearance (Figs. 10 and 11).

Twenty-five types of cis-acting elements were identified in the E2 genes, and 10 of them were selected to further study, which were auxin-, light-, abscisic acid-related elements, and core promoter elements around -30 of transcription start, as well as defense and stress-, low-temperature-, drought response-, circadian control-, and meristem expression-related elements, and, finally, seed-specific regulation-responsive elements. Light response and abscisic acid-responsive elements were the most widely distributed among the E2 genes, which were present in each E2 gene and with much higher numbers than the other elements. It is quite possible that almost all E2 genes might respond to the light and abscisic acid in *Gp. lemaneiformis*, and the differential expression of E2 genes during tetrasporophyte development might also be caused by these two abiotic factors. Core promoter elements around -30 of the transcription start, low-temperature-responsive, meristem expression-related, and circadian control elements were observed in 12, 11, 9, and 4 E2 genes, respectively. Auxin-responsive elements occurred in seven E2 genes, in which abscisic acid-responsive elements were also found. There were defense and stress-related elements in four genes: LXC005843.1, LXC006838.1, LXC006951.1, and LXC007427.1. Drought response-related elements were found in four E2 genes, accompanied by the existence of low-temperature- and meristem expression-related elements. The seed-specific regulation-responsive elements were present only in LXC006951.1 and LXC002619.2, and the types of elements contained in the gene LXC002619.2 were very similar to LXC006951.1 and LXC002619.1.

Twenty-six types of cis-acting elements were discovered in the E3 genes, and 10 of them were selected to further study, which were auxin-, light-, MeJA-, abscisic acid-, and gibberellin-responsive elements, as well as defense and stress-, low-temperature-, drought inducibility-, circadian control-, cell cycle regulation-responsive elements. Almost a quarter of the total number were light-responsive elements, such as Sp1, I-Box, G-Box, TCCC-motif, GT1-motif, TCT-motif and so on, which were contained in each E3 gene. It is quite possible that almost all E3 genes might respond to light during the growth and development process of *Gp. lemaneiformis*. Furthermore, there were also lots of MeJA-responsive elements in every E3 gene except *GlMIB2-1*(LXC000912.1). Auxin-responsive elements were found on 32 *Gp. lemaneiformis* E3 genes, drought-induced elements were found on 38 E3 genes, gibberellin-responsive elements were found on 32 E3 genes, and low-temperature-responsive elements were found on 39 E3 genes. These elements were distributed on almost all E3 genes, although they were few in number. In addition, a large number of abscisic acid-responsive elements were contained in 11 genes. The rest, including cell cycle regulation, defense and stress responsiveness, and circadian control elements, occurred on no more than 10 genes, and they were also few in number. Altogether, it was suggested that E3 genes in *Gp. lemaneiformis* might responded to various regulation pathways, particularly light response and phytohormone-responsive regulation because there were the most cis-acting elements of light- and phytohormone-responsive regulation.

### Expression pattern analysis of E3 genes at different stages of tetrasporophyte development

The *Gp. lemaneiformis* ‘981’ cultivar has difficulties in tetraspore release, releasing low numbers of tetraspores, and tetraspore deformity [62]. Strain ‘WLP-1’ releases tetraspores rapidly [63]. ZC and wild type (WT) have no abnormalities or special features in tetraspore release and were used as controls in our transcriptome analyses. The materials at different stages of tetrasporophyte development were subjected to transcriptome sequencing. The stages were: prophase of tetraspore formation (stage I), the period of tetraspore formation and release (combined stage II and III), and the recovery period after tetraspore release (stage IV) [55, 64].

**Fig. 12:**
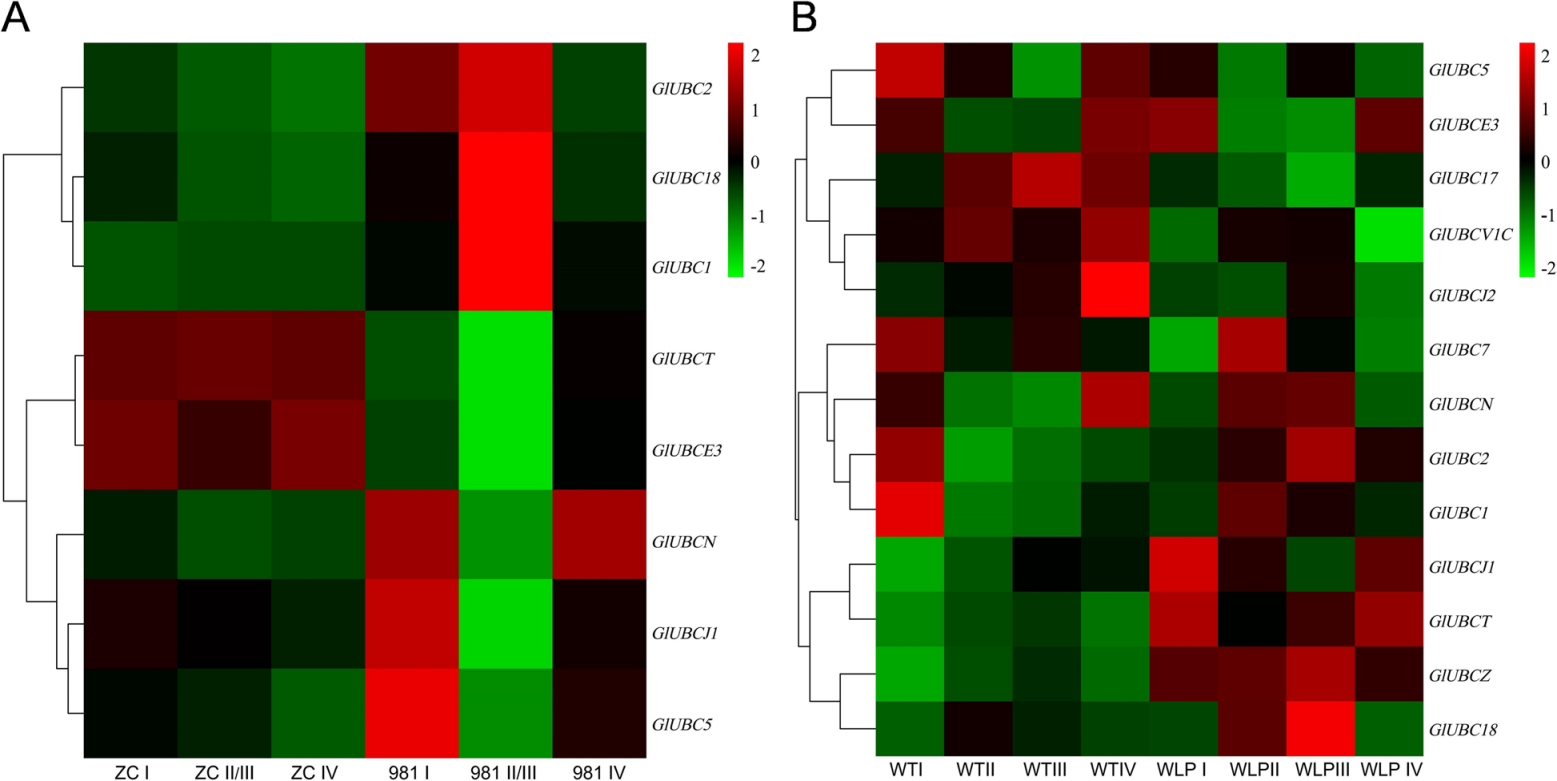
Analysis of the expression patterns of E2 genes in *Gp. lemaneiformis* during different stages of tetrasporophytes development. **A** The heatmap of 981 and ZC on three stages (stage II and III were merged) of tetrasporophytes development. **B** The heatmap of WLP-1 and WT on four stages of tetrasporophytes development. The color bar represents log_2_ expression levels (FPKM), and the lower expression of genes was shown with green shades as well as higher expression of genes was shown using red shades. The tree on the left represents clustering result of genes expression pattern

To better observe the potential function of E2 genes during the four stages of tetrasporophyte development, RNA-Seq data (SRR23946942 and SRR23949127) were analyzed (Fig. 12). Expression analysis of E2 genes in 981 and ZC revealed low (< 1.0) or no expression in five E2 genes of all treatments (Fig. 12A), while all E2 genes in WLP-1 and WT exhibited expression (Fig. 12B). At combined stages II and III in 981, the expression levels of these E2 genes were significantly different from that at stages I and IV, and this difference was greater than that in ZC. The same situation also existed in WLP-1 and WT. it was indicated that the E2 genes were generally up- or down-regulated during the process of tetraspores formation and release in *Gp. lemaneiformis*, especially in cultivar 981.

Among them, *UBCN* (LXC007561.1) was the only gene up-regulated at combined stage II and III in WLP-1 but down-regulated in the other three strains, which might be related to the type of cultivars/strains. In addition, seven genes had the same expression pattern in 981 and WLP-1: three genes, *UBC2*(LXC007342.1), *UBC18*(LXC004411.1), and *UBC1*(LXC001783.1), were up-regulated during stages II and III compared to stage I. Four genes, *UBCT* (LXC002092.1), *UBCE3*(LXC007823.1), *UBCJ1*(LXC007427.1), and *UBC5*(LXC002619), were down-regulated during stages II and III. The expression level of these seven genes above might only be related to stages of tetrasporophyte development. Moreover, five genes, *UBC17* (LXC006838.1), *UBCV1C* (LXC000128.1), *UBCJ2* (LXC005843.1), *UBC7* (LXC006951.1), and *UBCZ* (LXC000565.1) did not exist expression level in 981, and might be only related to the process of tetrasporophyte development in WLP-1.

**Fig. 13:**
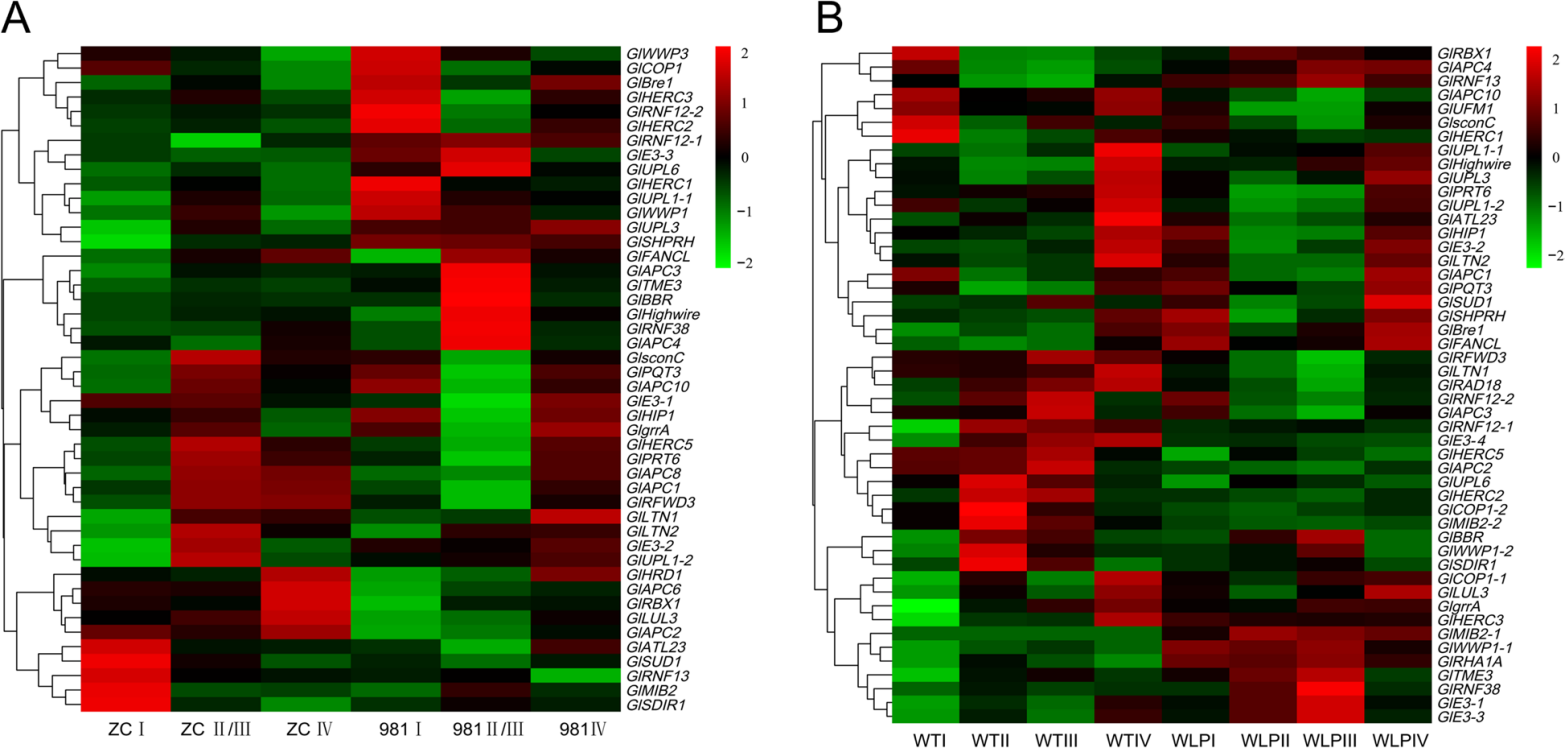
Analysis of the expression patterns of E3 genes in *Gp. lemaneiformis* during different stages of tetrasporophytes development. **A** The heatmap of 981 and ZC on three stages (stage II and III were merged) of tetrasporophytes development. **B** The heatmap of WLP-1 and WT on four stages of tetrasporophytes development. The color bar represents log_2_ expression levels (FPKM), and the lower expression of genes was shown with green shades as well as higher expression of genes was shown using red shades. The tree on the left represents clustering result of genes expression pattern

To study the potential rule of the E3 genes in regulating the process of tetraspore formation and release, two set of RNA-Seq data were analyzed (Fig. 13). The results showed that the expression levels of most E3 genes at stages II and III exhibited significant up- or down-regulation compared to that at stages I and IV, especially in 981 and WLP-1. This was very similar to the situation of E2 genes in various cultivars/strains.

According to the expression levels of these E3 genes, the expression patterns could be divided into several types. Firstly, there were similar expression pattern in low-fertility cultivar 981 and high-fertility strain WLP-1 of genes LXC001726, LXC000452, LXC000861, LXC005020, LXC004472, LXC003681, LXC003991, LXC000853, LXC000486, LXC006806, LXC007689, LXC000380, LXC006375, LXC006211, LXC007039 and LXC001652, which displayed both down- or up-regulation at stages II and III compared to that at stages I. In addition, there was an opposite expression pattern in 981 and WLP-1 of genes LXC007412, LXC008086, LXC000515, LXC004838, LXC000111, and LXC002035, which were up- (or down-) regulated in 981 and down- (or up-) regulated in WLP-1 at stages II and III compared to that at stages I. In addition, there were irregular expression trends of the genes LXC006100, LXC000862, LXC001227, and LXC007722 in different cultivars/strains. Most E3 genes in cultivars/strains with different fertility were significantly up-/down-regulated during the process of tetraspore formation and release, indicating that these *Gp. lemaneiformis* E3 ubiquitin ligases might be involved in the regulation of tetraspore release directly or indirectly.

**Fig. 14:**
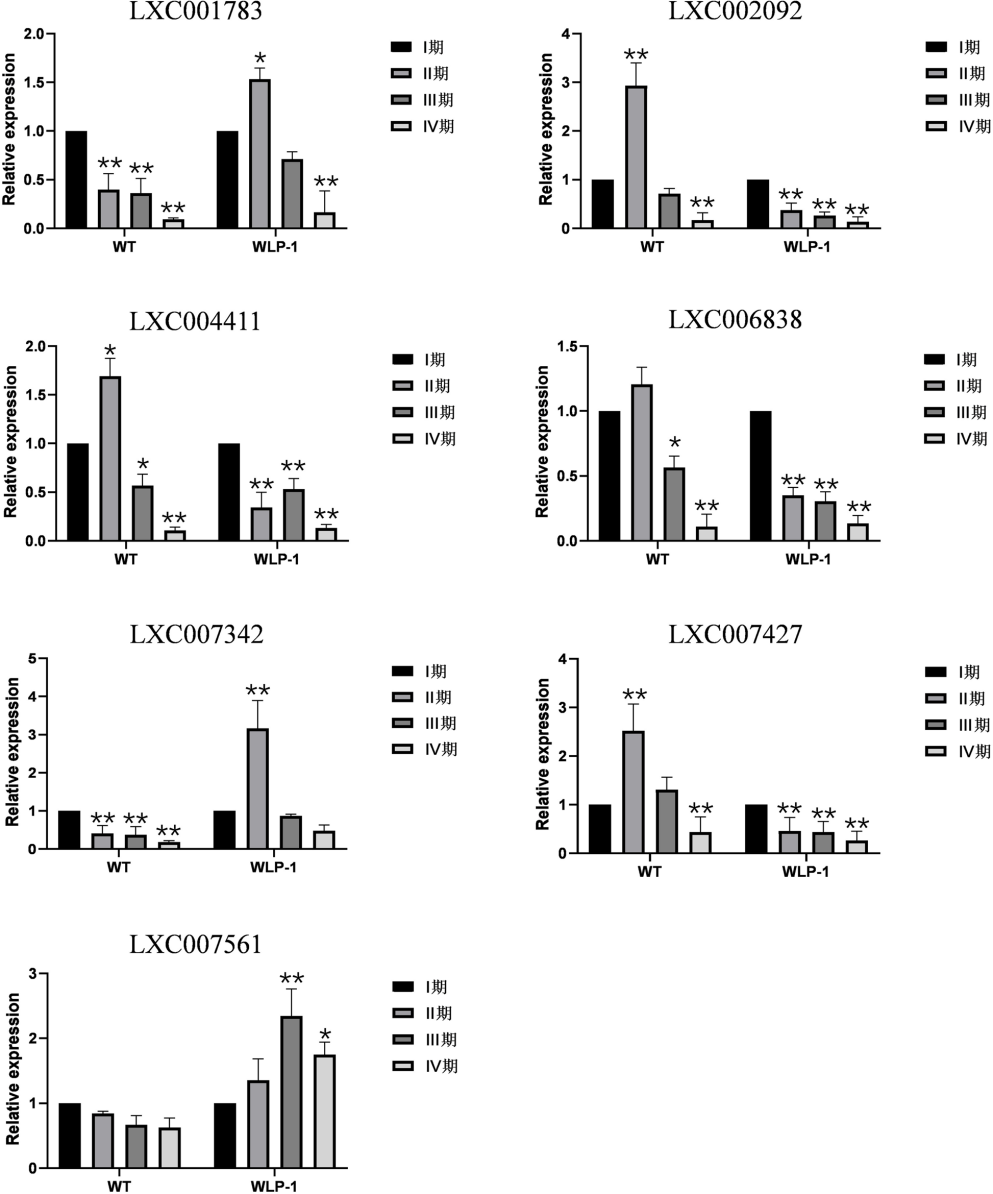
Gene expression level verification of E2 genes based on qRT-PCR. Error bars were standard deviations from the biologic replicates

**Fig. 15:**
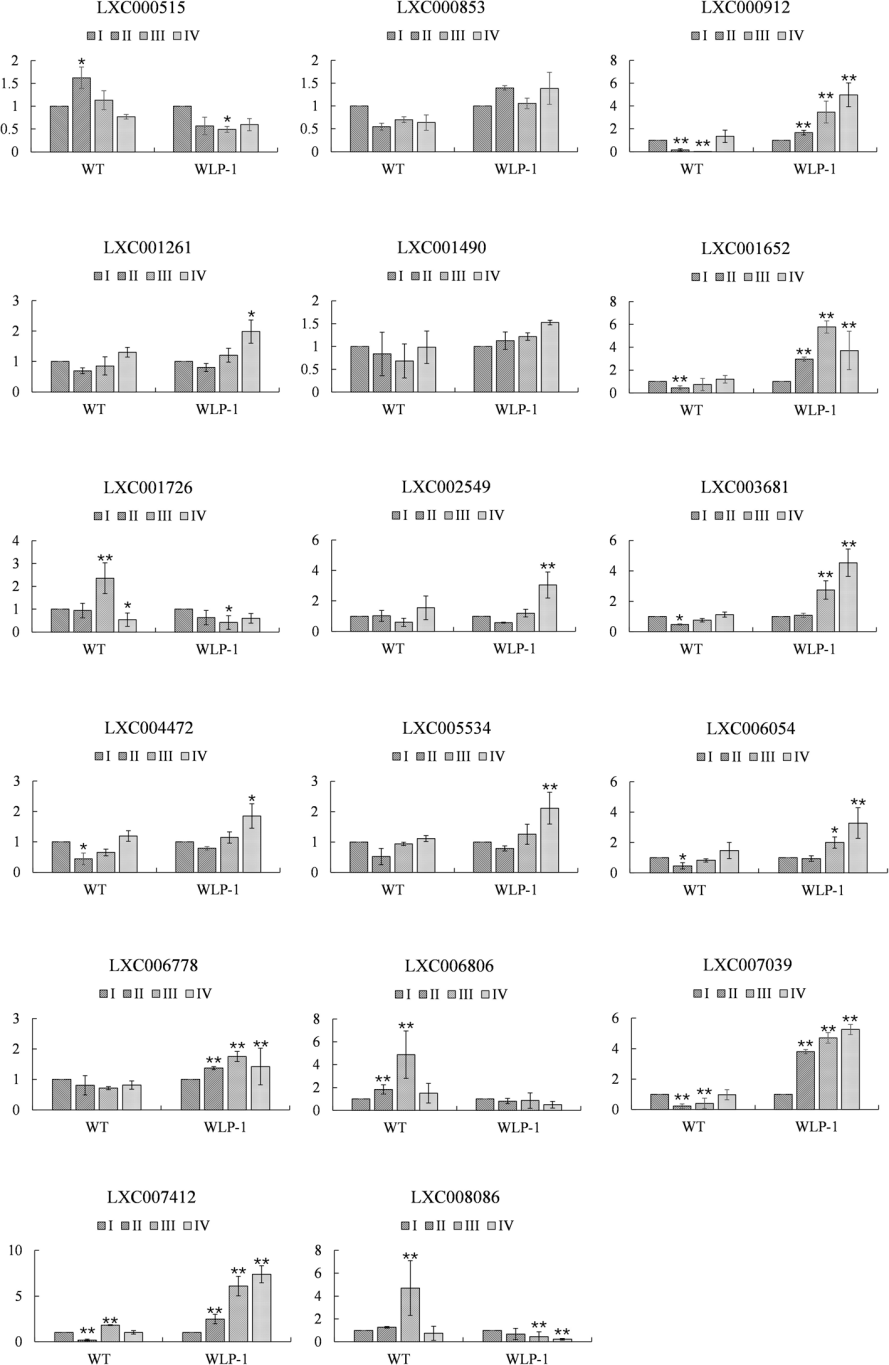
Gene expression level verification of E3 genes based on qRT-PCR. Error bars are standard deviations from the biologic replicates

Subsequently, these RNA-seq expression patterns of the genes were verified by qPCR with strains WLP-1 and WT as materials. *18 s* and *gapdh* as internal reference genes, seven E2 genes and 17 E3 genes were selected for a quantitative experiment (Figs. 14 and 15). The experimental results showed that most genes were highly consistent with the levels in heatmaps except *UBC18*(LXC004411.1), *UBCT* (LXC002092.1), *UBCJ1*(LXC007427.1) and *WWP1* (LXC006806), indicating the expression level determined by RNA-seq was reliable.

## Discussion

*Gracilariopsis lemaneiformis* is mainly used to extract agar and feed abalone in industry. Its life history is a process of generation alternation. In the sporophyte generation, tetraspore release leads to the injury of algae, affecting yield and quality. The UPS is extremely important for protein degradation in all known eukaryotic organisms. Both E2 ubiquitin conjugating enzyme and E3 ubiquitin ligase belong to the UPS. E2s are involved in many key processes, including the reaction to abiotic stress [65–67], growth, and development [68]. E3s determine the specific recognition of target proteins and play the most important role in ubiquitination [69]. Except for the study on the response to dehydration stress of some E3 ubiquitin ligases in *Gloiopeltis fulcate* [70], the role of E3 ligases in other red algae has not been reported. In this study, we comprehensively analyzed the characteristics of E3 ubiquitin ligases and their expression patterns during tetrasporophyte development in *Gp. lemaneiformis*.

### Evolutionary analysis of E2 and E3 genes

Fourteen E2 genes and 51 E3 genes were identified. All E2s belonged to the UBC family, which contained only one conserved domain, the UBC domain. The role of E2 genes in the evolution and family expansion was suggested to be specific and stable. E3 genes were divided into three superfamilies according to their conserved domains, namely the RING-type superfamily, HECT-type family, and APC/C family. It was shown by chromosome location analysis that E2 and E3 genes were evenly distributed on 28 chromosomes. Introns play an important role in alternative splicing and the generation of non-coding RNA [71], which is believed to provide evolutionary advantages and increase protein diversity through exon shuffling and alternative splicing [72]. Interestingly, it was shown by gene structure analysis that there were one to four introns in E2 genes, while there were no introns in most E3 genes. This may be due to the need of E3s to react rapidly during the process of ubiquitination, as E3 ubiquitin ligase is the last and most important link in ubiquitination. And it was also found that there was huge difference in the sizes of E3 genes. The differences in structure and size led to functional diversity. Therefore, the function of E3s in *Gp. lemaneiformis* should also be as diverse as E3s in high animals and plants, widely participating in the regulation of multiple biological processes and protein degradation [3, 31–49]. Motif analysis showed that there were similar motifs in genes with similar evolutionary relationships. Noting that many motifs only existed in specific groups was worthy of further study.

The E3 ubiquitin ligases of six other algae were also analyzed, and 224 E3 genes were identified. There was no direct relationship between genome size of the species and the number of E3 genes, as 51 E3 genes were identified in *Gp. lemaneiformis* (86.66 Mb, SRA: SRR20338037), 44 in *Agarophyton vermiculophylla* (87.66 Mb), 25 in *Chara braunii* (1.8 Gb, GenBank: gca_003427395.1), 33 in *Chondrus crispus* (104.8 Mb, GenBank: gca_000350225.2), 39 in *Chlamydomonas reinhardtii* (112.5 Mb, GenBank: gca_018257845.1), 49 in *Gp. chorda* (92.18 Mb, GenBank: gca_003194525.1), and 37 in *Porphyridium purpureum* (19.67 Mb, GenBank: gca_019702435.1). However, the similarity between orthologous genes was consistent with the evolutionary relationships between the species; that is, the evolution of the genes was consistent with the evolution of the species, which further confirmed the importance of E3 ubiquitin ligase.

HECT-type E3 ubiquitin ligases were all single-subunit proteins [73] and were divided into three subfamilies according to the conserved domain: NEDD4, WW, and HECT. The number of HECT-type E3 genes of six species (except *Chara braunii*) was basically the same, and the number of the three HECT subfamilies in *Gp. lemaneiformis* was also basically the same. The conserved domains of each APC/C-type member in *Gp. lemaneiformis* and the number of APC/C subunits in seven species were both different, which was consistent with that in higher organisms. For example, there were 11 APC/C subunits in *Arabidopsis thaliana*, and at least 19 in high animals. This result might be related to the better adaptation of species according to the evolutionary process. RING-type E3 ubiquitin ligases were the most abundant and differentiated E3s, and these were divided into zf-RING, U-box, Cullin and ZZ types. The number of RING-type E3 genes in seven species of algae was different, and the number of RING-type genes in *Gp. lemaneiformis* differed greatly, with 17 U-box type genes and only four ZZ-type genes.

### Cis-regulatory element analysis of E2 and E3 genes

The analysis of cis-regulatory elements showed that all E2 and most E3 gene promoters contained light-responsive elements, hormone-corresponding elements, and stress and defense response elements. It was speculated that E2 conjugating enzymes and E3 ubiquitin ligases in *Gp. lemaneiformis* might respond to photomorphogenesis, effect of phytohormones, and adaptation to environmental stress. Many E2 and E3 genes contained two or more identical cis-acting elements, which can enhance transcription regulation and adaptation to environmental changes [74].

### The role of E2 genes during tetrasporophyte development

Transcriptome sequencing of different *Gp. lemaneiformis* cultivars and strains at different stages of tetrasporophyte development was carried out. The expression levels of all E2 genes were analyzed by heatmaps, and seven genes were selected for qPCR. We found that the E2 genes in 981 showed the most significant up- or down-regulation during the process of tetrasporophte development. Among them, the expression of *UBCN* was only related to type differences of strains or cultivars, which displayed opposite expression patterns in low-fertility cultivar 981 and high-fertility strain WLP-1. The expression of some other genes was mainly related to the different tetrasporophyte development stages, which dispalyed similar expression patterns in low-fertility cultivar 981 and high-fertility strain WLP-1, including *UBC2*, *UBC18*, *UBC1*, *UBCT*, *UBCE3*, *UBCJ1*, and *UBC5*. These seven genes might make similar influence during tetraspore formation and release in both WLP-1 and 981*.* Five E2 genes, *UBC17*, *UBCV1C*, *UBCJ2*, *UBC7*, and *UBCZ*, were not expressed during stages II and III in 981, which might only influence the process of tetraspore formation and release in WLP-1.

### The role of E3 genes during tetrasporophyte development

The type of expression pattern of E3 genes varied from the cultivars/strains during the four stages of tetrasporophyte development, and we might pay more attention to the E3 ubiquitin ligases with similar or opposite expression patterns in low-fertility cultivar 981 and high-fertility strain WLP-1. On one hand, the genes with consistent expression patterns in different fertility-ability cultivars/strains might be expressed very conserved, and the changes in gene expression levels were only related to stages of tetrasporophyte development. This kind of genes was named ‘stages related gene’, such as *GlRNF38* (LXC000380.1), *GlRNF13* (LXC007039.1), *GlTME3* (LXC001652.1) and so on. On the other hand, the genes with opposing expression patterns in different fertility-ability cultivars/strains might be the key genes affecting different tetraspore release characteristics in cultivars and strains, and this kind of gene was called ‘cultivar/strain type related gene’, such as *GlAPC3* (LXC000515.1), *GlE3-1* (LXC002035.1) and so on.

According to KEGG enrichment analysis of two transcriptomes, 51 E3 genes were co-enriched for ubiquitin mediated proteolysis (ccp04120) [75–77] during tetraspore formation and release (Tables S3 and S4). Ten of 51 genes encoded E3 ubiquitin ligases (UPL3, APC2, APC1, APC8, APC3, RBX1, COP1, FANCL, HRD1 and LUL3). Two others, CDC20 and CDH1 (CDC20 homologue 1) were activators of the APC/C complex [78, 79]. We mapped the APC/C and its activator genes to the *Arabidopsis thaliana* genome to understand their possible functions. *GlAPC3* (LXC000515.1) matched two alleles of *CDC27a* (AT3G16320) and *CDC27b* (*HOBBIT*, AT2G20000). These two genes were necessary for post-embryonic cell division and root tissue differentiation, and there were redundancy and functional differences between *AtCDC27a* and *AtCDC27b* at different developmental stages [80]. *GlAPC1* (LXC003681.1) matched AT5G05560, which played a synergistic role with *GlAPC4* in female gametogenesis and embryogenesis [81]. *GlAPC2* (LXC001261.1) matched AT2G04660, which was the core and largest subunit of the APC/C complex [82]. *GlAPC8* (LXC007451.1) matched AT3G48150, which was necessary for male meiosis in *Arabidopsis* [83]. *GlCDC20* (LXC001292.1) matched the alleles *CDC20.1* (AT4G33270), *CDC20.2* (AT4G33260), *CDC20.3* (AT5G27080), *CDC20.4* (AT5G26900), and *CDC20.5* (AT5G27570). *GlCDH1* (LXC004385.1) matched the alleles *CCS52A1* (AT4G122910), *CCS52A2* (AT4G11920), and *CCS52B* (AT5G13840). Inactive APC/C needs to be activated by CDC20 and/or CCS52 (CDH1) before mediating proteolysis, and during the inactive period of APC/C, APC3 interacts with APC8 and APC10 [84]. To sum up, the APC/C complex plays an important role in regulating the cell cycle, meiosis, mitosis, and growth and development in *Arabidopsis thaliana*. Therefore, the APC/C, together with its activators, might play an important role in regulating tetrasporophyte development and tetraspore release in *Gp. lemaneiformis*. In particular, *GlAPC3* showed much higher expression levels in 981 and much lower expression levels in WLP-1 compared with other E3 genes.

### The important role of APC/C during tetraspore formation and release

**Fig. 16:**
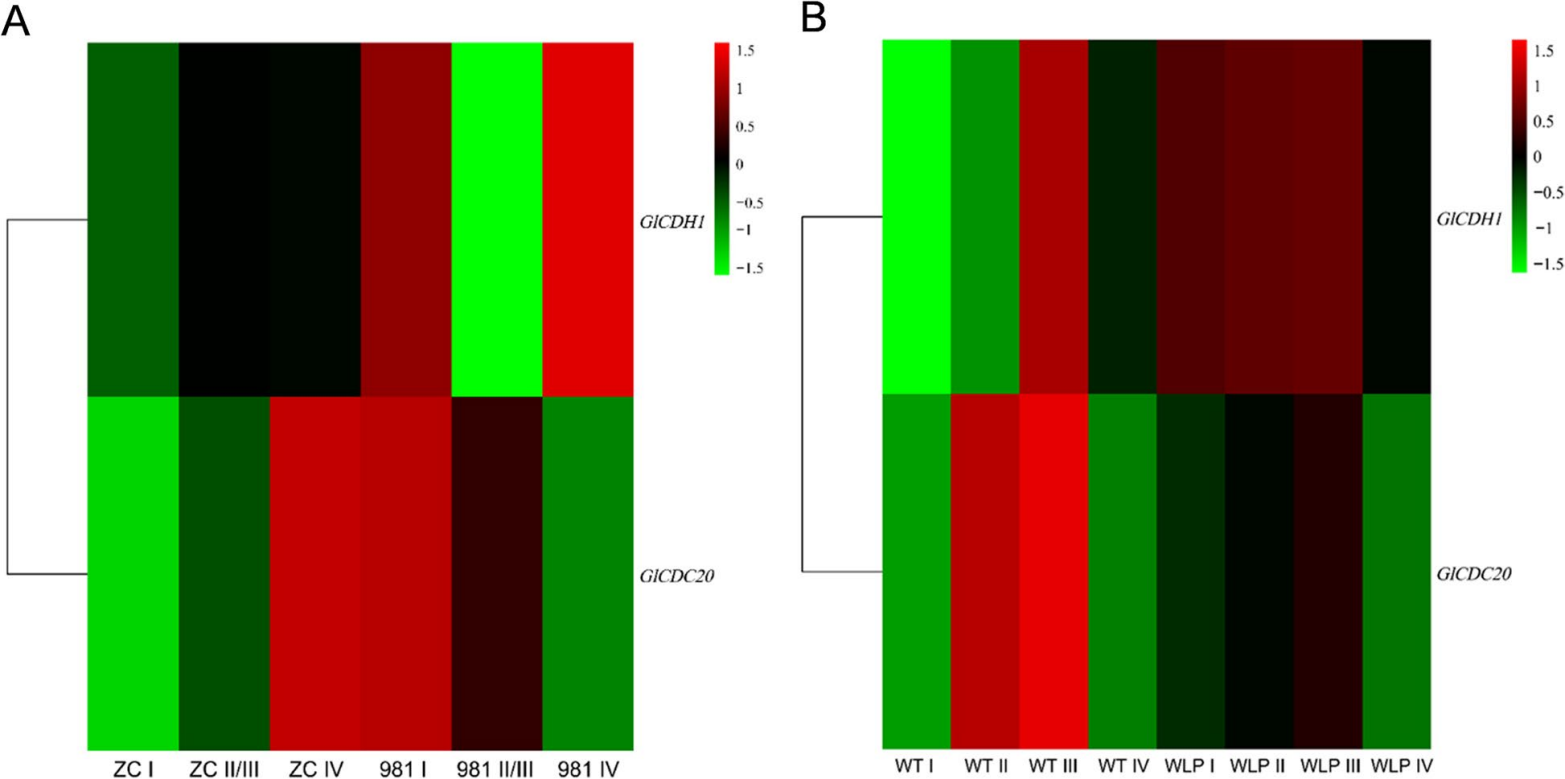
Expression patterns of *CDH1* and *CDC20* in *Gp. lemaneiformis* during different stages of tetrasporophytes development. The color bar represented log_2_ expression levels (FPKM). **A** Heatmap of *CDH1* and *CDC20* in cultivars 981 and ZC (stage II and III were merged). **B** Heatmap of *CDH1* and *CDC20* in strains WLP-1 and WT. The color bar represented log_2_ expression levels (FPKM), and the lower expression of genes was shown with green shades as well as higher expression of genes was shown using red shades. The tree on the left represented clustering result of genes expression pattern

**Fig. 17:**
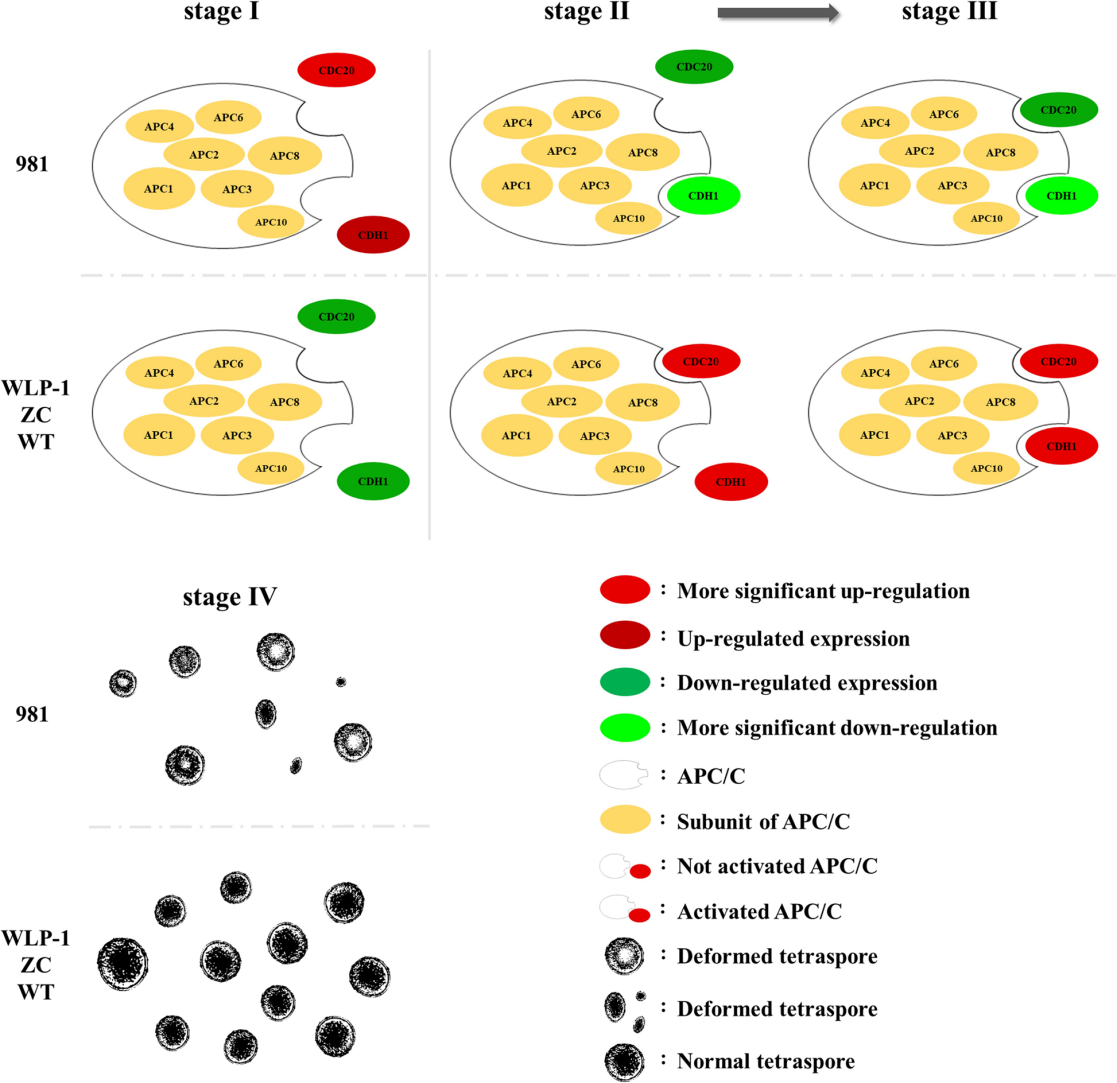
The putative regulation of APC/C ^CD20/CDH1^ during tetrasporophyte development in *Gp. lemaneiformis*

According to previous reports, the activators CDC20 and CDH1 of the APC/C existed in all known eukaryotic genomes [85]. Compared to the regulation of sister chromatids by the APC/C, the activation of the APC/C by CDC20 and CDH1 is an extremely rapid process [74]. Both of these co-activators bind to APC/C through C-box and Ile-Arg tail motifs [86]. As shown in Fig. 16, both *CDC20* and *CDH1* were up-regulated in ZC, WLP-1, and WT during stages II and III compared to stage I, indicating that CDC20 and CDH1 might activate the APC/C rapidly to promote tetraspore formation on stage II. Therefore, the expression of *CDC20* and *CDH1* was up-regulated at stage II and continued in stage III compared to stage I, and until the end of tetraspore release at stage IV, the gene expression decreased. In addition, according to the expression patterns of the two activators in three strains, except 981, it could be seen that the difference in expression levels of *CDC20* between stages II/III and stages I/IV was greater than that of CDH1. In 981, however, this difference in expression levels of *CDH1* was more significant. It has been proved in high plants and animals that there is a certain redundancy in the activation of these two activators, and only one activator plays the main role at any time [87–89]. Therefore, it was speculated that there might also be functional redundancy in *CDH1* and *CDC20* of *Gp. lemaneiformis*, and they might play the leading role at different times. That is, during tetraspore release in ZC, WT and WLP-1, *CDC20* might play a leading role in the activation of the APC/C and be rapidly up-regulated at stage II, earlier than the up-regulation of *CDH1*. The expression difference of *CDC20* between stage II and I in ZC, WT and WLP-1 might also be higher than that of *CDH1*. In 981, as the expression difference at stage II and I of *CDH1* was more significant than that of *CDC20*, *CDH1* might play a leading role in the activation of the APC/C. in addition, The *CDH1* and *CDC20* in 981 were down-regulated during tetraspores formation and release, while *CDH1* and *CDC20* in WLP-1, ZC, and WT were up-regulated during the time. The low fertility of cultivar 981 might be highly correlated with the inactivity of activators CDH1 and CDC20. Based on the above, the figure (Fig. 17) of regulation of APC/C^CD20/CDH1^ was drawn to help understand.

**Fig. 18:**
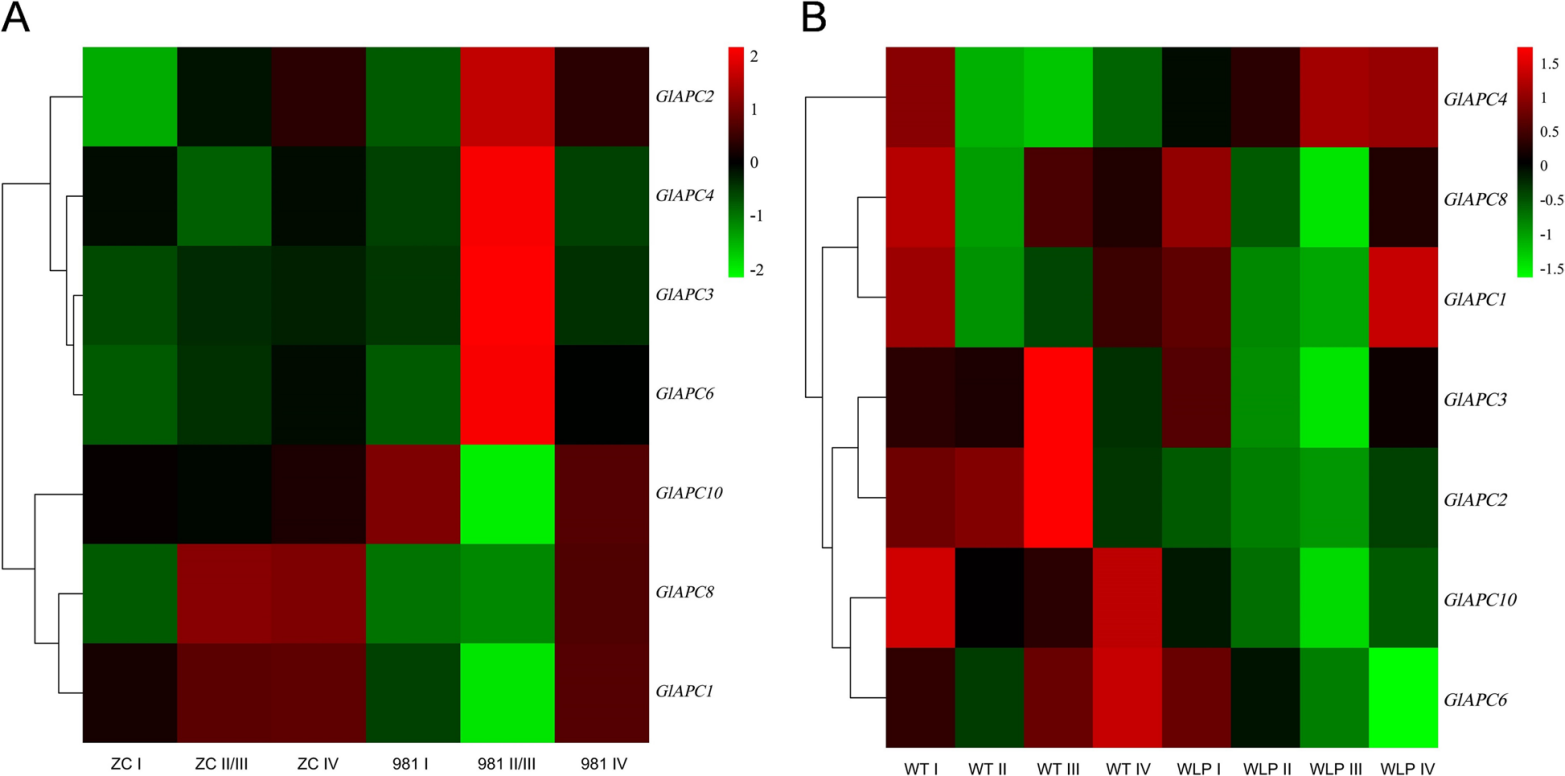
Expression patterns of APC/C subunits in *Gp. lemaneiformis* during different stages of tetrasporophytes development. **A** Heatmap of *CDH1* and *CDC20* in cultivars 981 and ZC (stage II and III were merged). **B** Heatmap of *CDH1* and *CDC20* in strains WLP-1 and WT. The color bar represented log_2_ expression levels (FPKM), and the lower expression of genes was shown with green shades as well as higher expression of genes was shown using red shades. The tree on the left represented clustering result of genes expression pattern

In WT and WLP-1 of *Gp. lemaneiformis* (Fig. 18B), the expression of all subunits of the APC/C, except APC2, were up-regulated at stage I, and when *CDC20* and *CDH1* were upregulated at stage II, the APC/C was rapidly activated and tetraspores were formed. After that, the expression level of the APC/C started to be down-regulated. In 981 (Fig. 18A), the expression levels of *APC10*, *APC8*, and *APC1* were the same as those in the WT, which also showed higher expression levels at stage I than at stages II and III. However, the expression levels of *APC2*, *APC4*, *APC3*, and *APC6* at stage I were significantly lower than at stages II and III. According to previous studies, APC2 and APC10 in humans are the catalytic cores of the APC/C [90, 91]. APC3 binds directly to CDC20 and CDH1 [80]. APC4 serves as a bridge for APC3 and APC1, to ensure the stability and function of the APC/C structure [92]. These four APC/C subunits are indispensable for receiving signals from CDC20 and CDH1. Therefore, it was speculated that the four proteins, APC2, APC4, APC3, and APC6, in *Gp. lemaneiformis* were more sensitive to signals from CDC20 and CDH1 than other APC/C subunits. Since both activators in 981 were down-regulated at stages II and III, the APC/C could not be activated, and the four proteins might enlarge the signals of chromosome separation and cell division through continuous synthesis, but chromosome separation and cell division promoted by the APC/C might still be difficult to complete due to changes in other genes or regulation mechanisms, resulting in extreme difficulty in releasing tetraspores and tetraspore deformation. In *Gp. lemaneiformis, APC10*, *APC8*, *APC4*, and *APC1* were ‘stages related gene’, and *APC2*, *APC3*, and *APC6* were ‘cultivars/strains type related gene’. Among them, the difference in the expression levels of *APC3* was the most significant, and it was enriched in ubiquitin mediated proteolysis. APC3 and APC7 bind directly to CDC20 and CDH1 in order to receive activating signals, making the APC/C active [80]. In summary, it was considered that APC3 might be the most important subunit of APC/C on regulation of the formation and release of tetraspores in *Gp. lemaneiformis* by ubiquitin mediated proteolysis.

## Conclusions

Fourteen E2 genes and fifty-one E3 genes were identified in *Gp. lemaneiformis*, and these were distributed on 11/ and 18/28 chromosomes, respectively. All E2s belonged to the UBC gene family, and the E3s were divided into 12 subgroups according to the conserved domains. The gene structures and phylogenetic analysis revealed the evolutionary history of the E2 and E3 genes in *Gp. lemaneiformis*. The patterns of motif distribution were relatively conserved in each family, except APC/C. The phylogenetic tree of seven species of algae indicated that the RING-type E3s showed the most functional differentiation. The expression levels of all E2 genes and most E3 genes, especially *GlUBCN* and *GlAPC3*, were significantly up-/ down-regulated during the process of tetraspore formation and release in *Gp. lemaneiformis*, especially in 981. *GlUBCN* was the only ‘cultivars/strains type related gene’ of the E2s, and *GlAPC3* was the ‘cultivars/strains type related gene’ with the most significant changes on expression level of E3s in *Gp. lemaneiformis*. *GlAPC3*, with *GlAPC1*, *GlAPC2* and *GlAPC8*, was involved in ubiquitin-mediated proteolysis of the APC/C and its activator genes *GlCDC20* and *GlCDH1*. *GlAPC3* showed a much higher expression level at stages II and III in cultivar 981 and a significantly decreased expression level at the same time in strain WLP-1. It was suggested that the E2 conjugating enzyme genes *GlUBCN* and *GlAPC3* might play an important role in tetraspore formation and release, and the activators *CDC20* and *CDH1* might activate the APC/C in *Gp. lemaneiformis* during tetraspore formation and release.

The results of this study provided a basic and comprehensive understanding of E2 and E3 genes in *Gp. lemaneiformis* and provided direction for further studying the regulatory mechanisms on tetraspore release in *Gp. lemaneiformis*.

## Materials and methods

### Algal materials and growth conditions

Four types of materials, cultivar 981 and ZC, as well as strain WLP-1 and wild type (WT) were used in our experiments. All strains mentioned above were preserved in the Key Laboratory of Marine Genetics and Breeding, Ocean University of China, and WT was collected from Zhanshan Bay (36°02’ N, 120°20’ E), Qingdao, China. Before experiments, they were all cultivated under 20 ± 1 °C for five days, with the 30 μmol·m^−2^·s^−1^ light intensity and 12hlight/12 h dark photoperiod [56].

Four stages samples were collected: prophase of tetraspores formation (stage I), period of tetraspores formation (stage II), period of tetraspores release (stage III) and recover period after tetraspores release (stage IV) [55, 64]. Samples were observed by an optical microscope every two days and four stages materials were collected with four biological repeats. All materials collected were quickly immersed in liquid nitrogen and frozen in -80 °C until the extraction of total RNA.

### Genome-wide sequence retrieval of E2 and E3 genes in Gracilariopsis lemaneiformis

The genome and protein sequences of E2 and E3 genes in *Gp. lemaneiformis* were found by annotation of our previous genome data (SRR20338037). To identify these candidate sequences, the Hidden Markov model (HMM) profile of the E2 and E3 conserved domain was respectively downloaded from the Pfam [93] (http://www.sanger.ac.uk/Software/Pfam/) database and then submitted as a query in a HMMER (e-value  < 1e^−5^) search (https://www.ebi.ac.uk/Tools/hmmer/) of the *Gp. lemaneiformis* protein database.

### Basic information analysis

Molecular weight, instability index, isoelectric point (pI) and grand average of hydropathicity (GRAVY) were analyzed by the online website ExPASy (http://web.expasy.org/protparam/).

### Phylogenetic analysis

Five phylogenetic trees were constructed and analyzed, including a phylogenetic tree of E2 ubiquitin conjugating enzymes in *Gp. lemaneiformis*, a phylogenetic tree of RING, HECT, and APC/C type E3 ubiquitin ligases in *Gp. lemaneiformis* separately, and a phylogenetic tree of E3 ubiquitin ligases of seven species which were *Agarophyton vermiculophylla*, *Chondrus crispus*, *Chara braunii*, *Chlamydomonas reinhardtii*, *Gracilariopsis chorda*, *Gp. lemaneiformis*, and *Porphyridium purpureum*.

Orthologs across the above algal species were investigated as follow steps: Firstly, all identified E3 genes of *Gp. lemaneiformis* were compared with the whole genome CDS sequences of other six algae species (e-value < 1e^−10^). Then, the selected homologous genes were analyzed conserved domains by NCBI CD-search (https://www.ncbi.nlm.nih.gov/cdd/) to further confirm the orthologs. In addition, genomes of the six algae species (except *Gp. lemaneiformis*) were screened again according to the genomic annotations to determine whether there were E3 genes that were not found by sequences alignment in the previous step, and conserved domains of these E3 genes were analyzed for further identification. Finally, all identified E3 genes were integrated into a total text. The trees of *Gp. lemaneiformis* were constructed by MEGA7.0 with the Maximum Likelihood (ML) method, and a bootstrap analysis was conducted using 1000 replicates with pairwise gap deletion mode. The protein sequences of other six species were all downloaded from the NCBI (https://www.ncbi.nlm.nih.gov/). All the sequences were aligned with the MAFFT program [94]. The phylogenetic tree was reconstructed by the Maximum Likelihood (ML) method implemented in IQ-TREE, with the best-fit model automatically selected by ModelFinder. Support for the inferred ML tree was obtained by ultrafast bootstrap approximation (UFBoot) with 1000 replicates [95, 96]. Figtree was used for visualization (http://tree.bio.ed.ac.uk/software/figtree/).

### Analysis of conserved motifs and gene structures

Conserved motifs contained in E2 ubiquitin conjugating enzymes and E3 ubiquitin ligase were predicted by MEME [97] (http://meme-suite.org/) online, and TBtools was used for visualization [98]. Also note that E3s needed to be predicted separately according to different gene family. The parameters of MEME were as follows: number of repetitions, any; maximum number of motifs, 10; and optimum motif widths, 6 to 200 amino acid residues.

Information of gene structures was from a GFF file, which was also the previous study of our team, and TBtools was used for visualization.

### Chromosomal location analysis

Chromosomal position information of all E2 and E3 genes were obtained from files of our *Gp. lemaneiformis* genome data (SRR20338037), and TBtools was used for visualization. The approximate steps of Chromosome localization of E2 and E3 genes was that the ‘LXC. Gff’ file (in the genome data of *Gp. lemaneiformis*) was input to Tbtools in the order of ‘Graphics—Show genes on chromosome—Gene location visualization from GTF/GFF’ to obtain relevant result.

### Promoter cis-acting element analysis

Nucleic acid sequence 2000 bp upstream of each candidate E3 gene was searched using TBtools and submitted to PlantCare (http://bioinformatice.psb.ugent.be/webtools/plantcare/html/) to predict the promoter cis-acting element. TBtools was also used for visualization. To better count the number of each element in each gene, a bar graph was constructed by excel software.

### RNA-seq

After *Gp. lemaneiformis* RNA extracted (RNeasy Plant Mini Kit, OMEGA), the sequencing work was carried out by Novogene® (Beijing, China). For specific methods of RNAseq, please refer to the paper previous published in our laboratory [57].

### Differential expression analysis

Differential expression analysis was performed using the DESeq2 R package (1.20.0) [99]. DESeq2 provide statistical routines for determining differential expression in digital gene expression data using a model based on the negative binomial distribution. Background genes whose expression levels were less than 1 in each group were filtered out. P-values were adjusted using the P-adjust [100, 101]. Genes were determined to be differentially expressed when the *P*-adjust ≤ 0.05 and |log2(FoldChange)|≥ 1.

### Analysis of expression levels of E2 and E3 genes during different stages of tetrasporophytes development

Heatmaps were drawn by the tool ‘heatmap’ of Omicshare platform (https://www.omicshare.com/tools/Home/Soft/heatmap) with default parameters, which based on the FPKM data from the RNA-seq dataset SRR23949127 (raised from 981 and ZC) and SRR23946942 (raised from WLP-1 and WT).

### The CDS sequences cloning of E2 and E3 genes

The full-length cDNA encoding E3 ubiquitin ligase genes were cloned. The primers were designed and synthesized according to the primer design software Primer Premier (Tables S5 and S6). The cDNA of wild type diploid was used as the template for PCR amplification. The PCR program was: 95 °C for 3 min, 95 °C for 15 s, 60 °C for 15 s, 30 s/kb at 72 °C, and cycle number of steps 2 to step 4 was 35, and 72 °C for 5 min at last. PCR products were subjected to agarose gel electrophoresis to be confirmed the size of the genes. Genes with the same size as the genome CDS sequence size were sent for sequencing.

### Quantitative real-time PCR (qRT-PCR) analysis

After *Gp. lemaneiformis* RNA extracted (RNeasy Plant Mini Kit, OMEGA) and the first-strand cDNA synthesized (HiScript III RT SuperMix for qPCR, Vazyme), quantitative real-time PCR (qRT-PCR) reaction was carried out according to the qPCR kit instructions (Vazyme, Nanjing, China) to validate the expression of each gene during various stages of tetraspores releasing. The specific gene primers for quantitative real-time PCR (qRT-PCR) were designed with Primer Premier 5 (Tables S7 and S8). The qRT-PCR program was divided into three stages: Stage 1 was 95 °C for 30 s, Stage 2 was 95 °C for 10 s, 60 °C for 20 s, and cycle number was 40. Stage 3 was 95 °C for 15 s, 60 °C for 60 s, and 95 °C for 10 s. In this experiment, per result consisted of four groups of biological repeats, each with four technical repetitions. Reference genes were *18S* (18 s rRNA) and *GAPDH* (encoding glyceraldehyde-3-phosphate dehydrogenase) [102], and the relative expression levels of the E3 genes were calculated by the methods of 2 ^−ΔΔCt^ [103, 104].

### Supplementary Information

**Additional file 1:** **Supplementary Fig. S1.** PCR amplification products of 13 E2 genes of wild type of *Gp. lemaneiformis*. M: marker. Number 1-13 indicated different E2 ubiquitin activating enzyme genes, and the left were DNA sequences, and the right were cDNA sequences.

**Additional file 2:** **Supplementary Fig. S2.** PCR amplification products of 48 E3 genes’ cDNA of wild type of *Gp. lemaneiformis*. M: marker. Number 1-48 indicated cDNA sequences of different E3 ubiquitin ligase genes.

**Additional file 3:** **Supplementary Fig. S3. **Conserved domains of E2 ubiquitin conjugating enzymes in *Gp. lemaneiformis*. **A** The phylogenetic tree. **B** Protein conserved domains.

**Additional file 4:** **Supplementary Fig. S4.** Conserved domains of HECT type E3 ubiquitin ligases in *Gp. lemaneiformis*.**A** The phylogenetic tree. **B** Protein conserved domains.

**Additional file 5:** **Supplementary Fig. S5.** Conserved domains of APC/C type E3 ubiquitin ligases in *Gp. lemaneiformis*. **A **The phylogenetic tree. **B** Protein conserved domains.

**Additional file 6:** **Supplementary Fig. S6.** Conserved domains of RING type E3 ubiquitin ligases in *Gp. lemaneiformis*. **A** The phylogenetic tree. **B** Protein conserved domains.

**Additional file 7:** **Supplementary Table S1.** List of 14 E2 ubiquitin conjugating enzymes genes identified in *Gp. lemaneiformis.*

**Additional file 8:** **Supplementary Table S2.** List of 51 E3 ubiquitin ligases genes identified in *Gp. lemaneiformis.*

**Additional file 9:** **Supplementary Table S3. **Genes of ubiquitin mediated proteolysis in cultivars 981 and ZC.

**Additional file 10:** **Supplementary Table S4. **Genes of ubiquitin mediated proteolysis in strains WLP-1 and WT.

**Additional file 11:** **Supplementary Table S5. **List of primer sequences for E2 genes DNA and cDNA sequences in *Gp. lemaneiformis.*

**Additional file 12:** **Supplementary Table S6. **List of primer sequences for E3 genes cDNA sequences in *Gp. lemaneiformis.*

**Additional file 13:** **Supplementary Table S7.** List of primer sequences for qPCR of E2 genes in *Gp. lemaneiformis.*

**Additional file 14:** **Supplementary Table S8.** List of primer sequences for qPCR of E3 genes in *Gp. lemaneiformis.*

## Data Availability

Raw sequencing data for all samples analyzed in this study have been uploaded to the National Center for Biotechnology Information (NCBI), and are available under accession number: SRR20338037, SRR23946942 and SRR23949127. All data generated or analyzed during this study are included in this published article (and its supplementary information files). Other information requested reasonably are available from Qiong Wu and requires permission from Zhenghong Sui.

## Abbreviations

APC/C: Anaphase promoting complex or cyclosome
UPS: Ubiquitin proteasome system
E1: Ubiquitin activating enzyme
E2: Ubiquitin conjugating enzyme
E3: Ubiquitin ligase
RING: Really Interesting New Gene
HECT: Homologous to E6-AP Carboxyl Terminus
RBR: RING between RING
HERC: HECT and RCC1-like domain
pI: Isoelectric point
GRAVY: Grand average of hydropathicity
