# Supplementary material for: Identification, characterization and expression profiles of E2 and E3 gene superfamilies during the development of tetrasporophytes in Gracilariopsis lemaneiformis (Rhodophyta)
Source: BMC Genomics. 2023 Sep 18;24:549. doi: 10.1186/s12864-023-09639-0 (PMC10506303; doi:10.1186/s12864-023-09639-0)
Supplement: Supplementary file 1 — Additional file 1: Supplementary Fig. S1. PCR amplification products of 13 E2 genes of wild type of Gp. lemaneiformis. M: marker. Number 1-13 indicated different E2 ubiquitin activating enzyme genes, and the left were DNA sequences, and the right were cDNA sequences. [file 12864_2023_9639_MOESM1_ESM.docx]

**
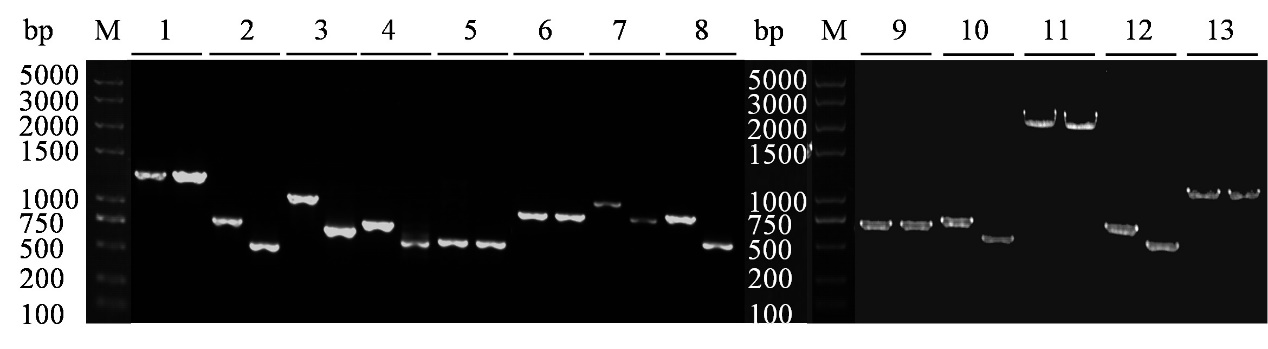
**

**Supplementary Fig. S1** PCR amplification products of 13 E2 genes’ cDNA of wild type of *Gp. lemaneiformis*. M: marker. Number 1-13 indicated different E2 ubiquitin activating enzyme genes, and the left were DNA sequences, and the right were cDNA sequences.
