# Supplementary material for: Identification, characterization and expression profiles of E2 and E3 gene superfamilies during the development of tetrasporophytes in Gracilariopsis lemaneiformis (Rhodophyta)
Source: BMC Genomics. 2023 Sep 18;24:549. doi: 10.1186/s12864-023-09639-0 (PMC10506303; doi:10.1186/s12864-023-09639-0)
Supplement: Supplementary file 2 — Additional file 2: Supplementary Fig. S2. PCR amplification products of 48 E3 genes’ cDNA of wild type of Gp. lemaneiformis. M: marker. Number 1-48 indicated cDNA sequences of different E3 ubiquitin ligase genes. [file 12864_2023_9639_MOESM2_ESM.docx]

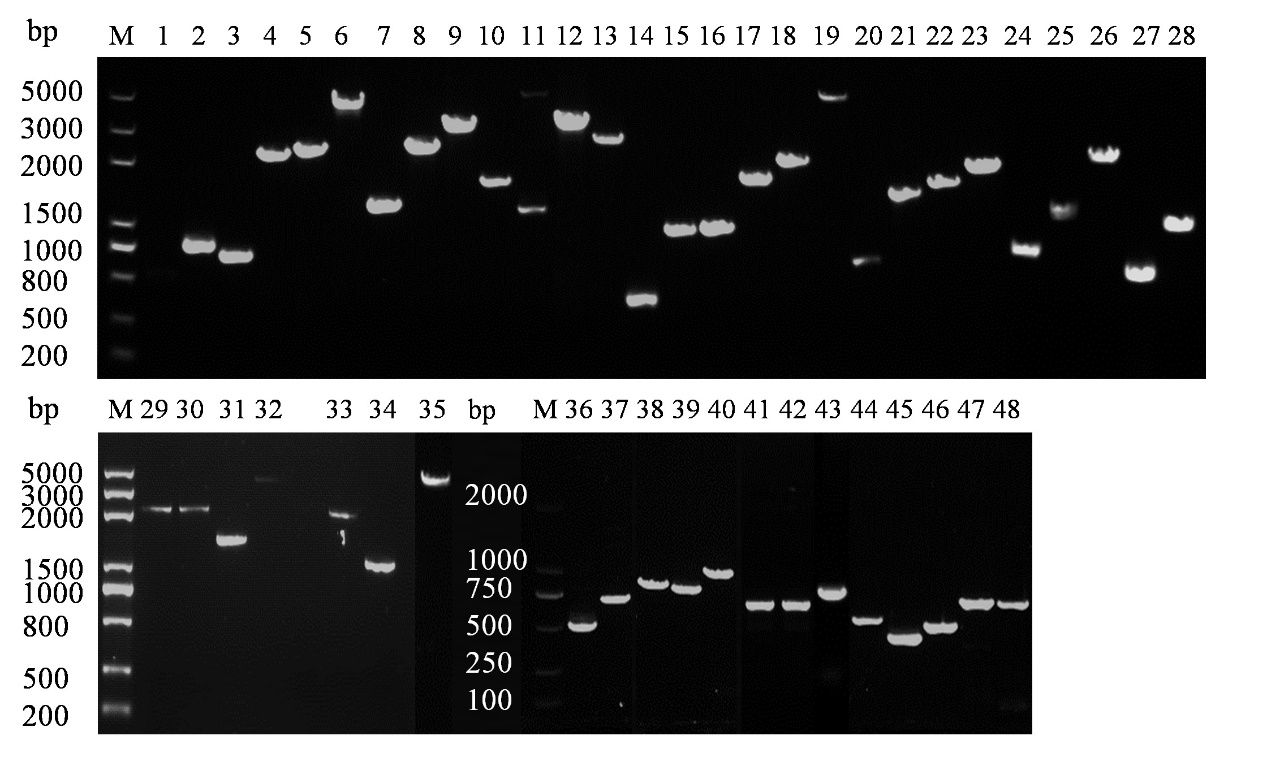


**Supplementary Fig. S2** PCR amplification products of 48 E3 genes’ cDNA of wild type of *Gp. lemaneiformis*. M: marker. Number 1-48 indicated cDNA sequences of different E3 ubiquitin ligase genes.
