# Supplementary material for: Identification, characterization and expression profiles of E2 and E3 gene superfamilies during the development of tetrasporophytes in Gracilariopsis lemaneiformis (Rhodophyta)
Source: BMC Genomics. 2023 Sep 18;24:549. doi: 10.1186/s12864-023-09639-0 (PMC10506303; doi:10.1186/s12864-023-09639-0)
Supplement: Supplementary file 3 — Additional file 3: Supplementary Fig. S3. Conserved domains of E2 ubiquitin conjugating enzymes in Gp. lemaneiformis. A The phylogenetic tree. B Protein conserved domains. [file 12864_2023_9639_MOESM3_ESM.docx]

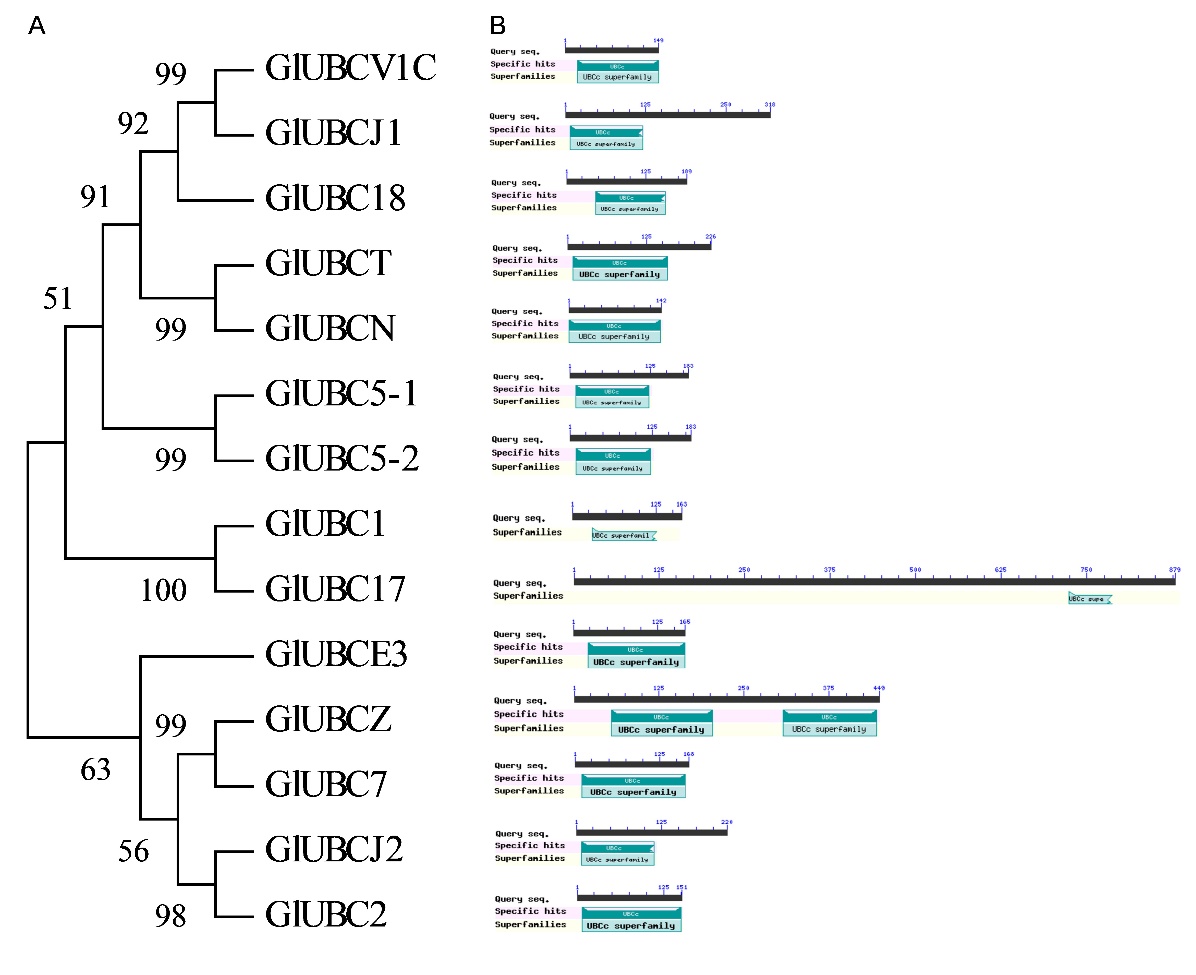


**Supplementary Fig. S3** Conserved domains of E2 ubiquitin conjugating enzymes in *Gp. lemaneiformis*. **A** The phylogenetic tree. **B** Protein conserved domains.
