# Supplementary material for: Identification, characterization and expression profiles of E2 and E3 gene superfamilies during the development of tetrasporophytes in Gracilariopsis lemaneiformis (Rhodophyta)
Source: BMC Genomics. 2023 Sep 18;24:549. doi: 10.1186/s12864-023-09639-0 (PMC10506303; doi:10.1186/s12864-023-09639-0)
Supplement: Supplementary file 4 — Additional file 4: Supplementary Fig. S4. Conserved domains of HECT type E3 ubiquitin ligases in Gp. lemaneiformis.A The phylogenetic tree. B Protein conserved domains. [file 12864_2023_9639_MOESM4_ESM.docx]

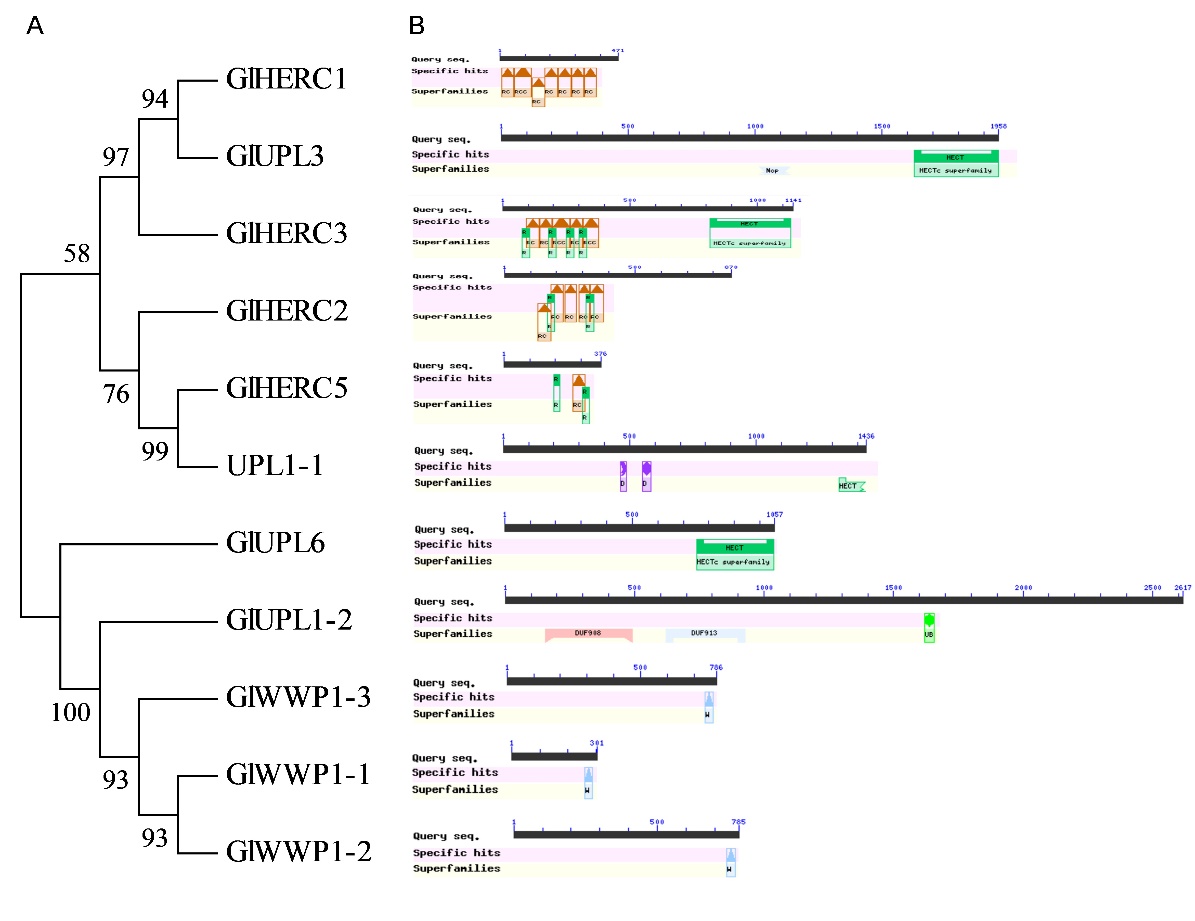


**Supplementary Fig. S4** Conserved domains of HECT type E3 ubiquitin ligases in *Gp. lemaneiformis*. **A** The phylogenetic tree. **B** Protein conserved domains.
