# Supplementary material for: Identification, characterization and expression profiles of E2 and E3 gene superfamilies during the development of tetrasporophytes in Gracilariopsis lemaneiformis (Rhodophyta)
Source: BMC Genomics. 2023 Sep 18;24:549. doi: 10.1186/s12864-023-09639-0 (PMC10506303; doi:10.1186/s12864-023-09639-0)
Supplement: Supplementary file 7 — Additional file 7: Supplementary Table S1. List of 14 E2 ubiquitin conjugating enzymes genes identified in Gp. lemaneiformis. [file 12864_2023_9639_MOESM7_ESM.docx]

**Supplementary Table S1.** List of 14 E2 ubiquitin activating enzymes genes identified in *Gp. lemaneiformis*

|  |  | | |  | | |  |  | |  | | | **Protein** | | | | |  | | |  | |  |
| --- | --- | --- | --- | --- | --- | --- | --- | --- | --- | --- | --- | --- | --- | --- | --- | --- | --- | --- | --- | --- | --- | --- | --- |
| **Name** | **Gene ID** | | **Chr** | | | **Location coordinates(N→C)** | | | **Gene length(bp)** | | **Extron** | | **pI** | **Length**  **(aa)** | | | **MW (Da)** | | | **Instability index** | | **GRAVY** | |
| GlUBC17 | | LXC006838.1 | | | 21 | | 1879759-1882728 | 2640 | | 2 | | 5.11 | | | 879 | 96515.7 | | | 52.28 | | -0.428 | |  |
| GlUBCJ1 | | LXC007427.1 | | | 24 | | 497803-498759 | 957 | | 1 | | 9.57 | | | 318 | 34818.2 | | | 68.57 | | -0.557 | |  |
| GlUBCV1C | | LXC000128.1 | | | 1 | | 891533-892235 | 703 | | 3 | | 7.86 | | | 149 | 16827.9 | | | 61.26 | | -0.574 | |  |
| GlUBC2 | | LXC007342.1 | | | 23 | | 1522534-1523154 | 621 | | 3 | | 5.39 | | | 151 | 17089.3 | | | 60.56 | | -0.556 | |  |
| GlUBCZ | | LXC000565.1 | | | 1 | | 906040-907389 | 1350 | | 1 | | 8.88 | | | 449 | 50625.4 | | | 48.9 | | -0.521 | |  |
| GlUBC1 | | LXC001783.1 | | | 4 | | 1757198-1757834 | 637 | | 3 | | 8.36 | | | 163 | 18969.8 | | | 38.54 | | -0.540 | |  |
| GlUBC5-1 | | LXC002619.1 | | | 6 | | 3111846-3112903 | 1058 | | 5 | | 4.32 | | | 209 | 23422.9 | | | 43.06 | | -0.655 | |  |
| GlUBC5-2 | | LXC002619.2 | | | 6 | | 3111846-3112903 | 1058 | | 5 | | 4.32 | | | 209 | 23422.9 | | | 43.06 | | -0.655 | |  |
| GlUBCE3 | | LXC007823.1 | | | 25 | | 1371760-1372473 | 714 | | 3 | | 5.76 | | | 165 | 18185.5 | | | 46.31 | | -0.294 | |  |
| GlUBCN | | LXC007561.1 | | | 24 | | 1294904-1295423 | 520 | | 2 | | 5.86 | | | 142 | 16170.5 | | | 57.39 | | -0.457 | |  |
| GlUBCT | | LXC002092.1 | | | 4 | | 3485887-3486567 | 681 | | 1 | | 6.06 | | | 226 | 25191.2 | | | 48.75 | | -0.738 | |  |
| GlUBC7 | | LXC006951.1 | | | 22 | | 626853-627683 | 831 | | 4 | | 5.05 | | | 168 | 19008.6 | | | 48.66 | | -0.455 | |  |
| GlUBC18 | | LXC004411.1 | | | 13 | | 796510-797079 | 570 | | 1 | | 8.56 | | | 207 | 23141.4 | | | 56.95 | | -0.343 | |  |
| GlUBCJ2 | | LXC005843.1 | | | 19 | | 9423624-9424502 | 879 | | 3 | | 10.01 | | | 220 | 25348.2 | | | 52.83 | | -0.426 | |  |
