# Supplementary material for: Identification, characterization and expression profiles of E2 and E3 gene superfamilies during the development of tetrasporophytes in Gracilariopsis lemaneiformis (Rhodophyta)
Source: BMC Genomics. 2023 Sep 18;24:549. doi: 10.1186/s12864-023-09639-0 (PMC10506303; doi:10.1186/s12864-023-09639-0)
Supplement: Supplementary file 8 — Additional file 8: Supplementary Table S2. List of 51 E3 ubiquitin ligases genes identified in Gp. lemaneiformis. [file 12864_2023_9639_MOESM8_ESM.docx]

**Supplementary Table S2.** List of 51 E3 ubiquitin ligases genes identified in *Gp. lemaneiformis*

| **Name** | **Gene ID** | **Chr** | **Location coordinates(N→C)** | **Gene length(bp)** | **Extron** | **Protein** | | | **Instability index** | **GRAVY** |
| --- | --- | --- | --- | --- | --- | --- | --- | --- | --- | --- |
|  |  |  |  |  |  | **pI** | **Length（aa）** | **MW (Da)** |  |  |
| GlAPC8 | LXC007451.1 | 24 | 593844-595202 | 1359 | 1 | 6.10 | 452 | 53093.5 | 38.64 | -0.567 |
| GlHERC5 | LXC000861.1 | 2 | 2940406-2941635 | 1230 | 2 | 9.24 | 376 | 40792.8 | 33.83 | -0.212 |
| GlAPC3 | LXC000515.1 | 2 | 705468-707585 | 2118 | 1 | 8.94 | 705 | 77421.9 | 45.32 | -0.356 |
| GlBBR | LXC006211.1 | 19 | 2382529-2383029 | 501 | 1 | 7.17 | 166 | 18523.9 | 56.03 | -0.41 |
| GlsconC1 | LXC005534.1 | 17 | 1265548-1266036 | 489 | 1 | 6.33 | 162 | 17866.1 | 54.75 | -0.683 |
| GlsconC2 | LXC005534.2 | 17 | 1265176-1266036 | 861 | 1 | 8.95 | 286 | 31987.5 | 57.75 | -0.967 |
| GlsconC3 | LXC005534.3 | 17 | 1264183-1266036 | 1854 | 1 | 9.34 | 617 | 69738.0 | 55.25 | -0.925 |
| GlAPC1 | LXC003681.1 | 10 | 506169-511532 | 5364 | 1 | 7.81 | 1787 | 197012.3 | 42.62 | -0.12 |
| GlPQT3 | LXC003991.1 | 10 | 2741102-2744168 | 3067 | 2 | 10.36 | 957 | 108109.6 | 77.05 | -1.488 |
| GlE3-1 | LXC002035.1 | 4 | 3117910-3118365 | 456 | 1 | 5.18 | 151 | 16680.7 | 53.32 | -0.473 |
| GlE3-3 | LXC006375.1 | 20 | 1133387-1134568 | 1182 | 1 | 6.30 | 393 | 44385.0 | 69.97 | -0.025 |
| GlATL23 | LXC002549.1 | 6 | 2660561-2660977 | 417 | 1 | 5.03 | 138 | 15167.6 | 60.09 | -0.015 |
| GlBre1 | LXC001490.1 | 3 | 2358952-2361626 | 2675 | 4 | 6.94 | 765 | 86120.8 | 50.49 | -0.79 |
| GlAPC10 | LXC006054.1 | 19 | 1571631-1572518 | 888 | 1 | 5.62 | 295 | 33786.7 | 35.18 | -0.823 |
| GlCOP1 | LXC006344.1 | 20 | 969690-971735 | 2046 | 1 | 8.84 | 681 | 75758.6 | 53.40 | -0.502 |
| GlFANCL | LXC007722.1 | 25 | 662162-663445 | 1284 | 2 | 5.23 | 398 | 45622.1 | 50.23 | -0.276 |
| GlHERC2 | LXC001726.1 | 4 | 970017-972698 | 2682 | 2 | 7.34 | 870 | 95262.5 | 43.75 | -0.11 |
| GlHighwire | LXC002149.1 | 4 | 3816108-3817502 | 1413 | 1 | 6.69 | 470 | 53056.3 | 50.85 | -0.268 |
| GlLTN1 | LXC005020.1 | 15 | 1391377-1392606 | 1230 | 1 | 9.66 | 409 | 46144.7 | 54.76 | -0.67 |
| GlLTN2 | LXC004838.1 | 14 | 2505900-2510453 | 4554 | 1 | 5.59 | 1517 | 166774.8 | 44.32 | -0.04 |
| GlMIB2-1 | LXC000912.1 | 2 | 3233747-3235252 | 1506 | 1 | 6.55 | 501 | 55675.9 | 40.38 | -0.469 |
| GlMIB2-2 | LXC007412.1 | 24 | 173427-175009 | 1583 | 2 | 8.95 | 481 | 53194.3 | 51.90 | -0.437 |
| GlMIB2-3 | LXC007412.2 | 24 | 173427-175009 | 1583 | 2 | 8.96 | 497 | 55040.5 | 51.94 | -0.432 |
| GlPRT6 | LXC000853.1 | 2 | 2914104-2920157 | 6054 | 1 | 6.69 | 2017 | 229668.6 | 45.40 | -0.281 |
| GlRBX1 | LXC001417.1 | 3 | 1555864-1556358 | 495 | 3 | 6.04 | 109 | 12456.2 | 42.92 | -0.50 |
| GlRFWD3 | LXC004472.1 | 13 | 1008376-1010271 | 1896 | 1 | 8.83 | 631 | 68951.0 | 51.51 | -0.393 |
| GlRNF12-1 | LXC001227.1 | 3 | 695931-696647 | 717 | 1 | 7.69 | 238 | 27243.1 | 83.69 | -0.582 |
| GlRNF12-2 | LXC000452.1 | 2 | 277187-277762 | 576 | 2 | 5.61 | 149 | 16390.2 | 62.30 | 0.204 |
| GlRNF13 | LXC007039.1 | 22 | 1254989-1255673 | 685 | 2 | 6.54 | 192 | 21702.3 | 72.18 | -0.748 |
| GlRNF38 | LXC000380.1 | 1 | 7289795-7291855 | 2061 | 2 | 9.81 | 637 | 70246.8 | 77.25 | -0.714 |
| GlSDIR1 | LXC006083.1 | 19 | 1657886-1658896 | 1011 | 1 | 5.74 | 336 | 37235.8 | 54.59 | -0.47 |
| GlSHPRH | LXC000111.1 | 1 | 835859-840742 | 4884 | 1 | 7.83 | 1627 | 183776.3 | 48.09 | -0.405 |
| GlUPL1-1 | LXC008198.1 | 27 | 1785507-1790053 | 4547 | 2 | 5.96 | 1436 | 160695.5 | 61.37 | -0.617 |
| GlUPL1-2 | LXC000862.1 | 2 | 2942255-2950261 | 8007 | 2 | 6.04 | 2617 | 285536.2 | 45.93 | -0.279 |
| GlUPL3 | LXC002801.1 | 7 | 1392776-1398652 | 5877 | 1 | 6.24 | 1958 | 215142.6 | 56.81 | -0.43 |
| GlUPL6 | LXC008086.1 | 27 | 1070045-1073218 | 3174 | 1 | 8.79 | 1057 | 117993.2 | 46.97 | -0.138 |
| GlHRD1 | LXC000691.2 | 2 | 1746759-1749254 | 2496 | 1 | 6.61 | 831 | 94186.6 | 56.48 | -0.528 |
| GlAPC6 | LXC000426.1 | 1 | 7573688-7575468 | 1781 | 2 | 8.31 | 560 | 62288.0 | 48.83 | -0.234 |
| GlWWP1-1 | LXC006806.1 | 21 | 1689912-1690817 | 906 | 1 | 5.06 | 301 | 33259.8 | 57.68 | -1.073 |
| GlWWP1-2 | LXC006806.2 | 21 | 1689912-1692269 | 2358 | 1 | 6.34 | 785 | 87658.5 | 54.46 | -0.754 |
| GlAPC2 | LXC001261.1 | 3 | 856390-858684 | 2295 | 1 | 5.63 | 764 | 87085.9 | 47.73 | -0.488 |
| GlWWP1-3 | LXC006812.1 | 21 | 1780487-1782847 | 2361 | 1 | 6.10 | 786 | 88045.0 | 57.89 | -0.766 |
| GlSUD1 | LXC007042.1 | 22 | 1557398-1560766 | 3369 | 1 | 4.62 | 1122 | 125648.4 | 39.28 | 0.048 |
| GlE3-2 | LXC006100.1 | 19 | 1698369-1714537 | 16169 | 5 | 5.88 | 5172 | 574287.3 | 49.49 | -0.221 |
| GlHERC1 | LXC000754.1 | 2 | 2094674-2096089 | 1416 | 1 | 5.86 | 471 | 50998.4 | 21.64 | -0.217 |
| GlHERC3 | LXC000422.1 | 1 | 7554253-7557678 | 3426 | 1 | 5.90 | 1141 | 126198.0 | 37.26 | -0.064 |
| GlHIP1 | LXC000486.1 | 2 | 417121-417720 | 600 | 1 | 8.43 | 199 | 22151.7 | 55.64 | 0.02 |
| GlLUL3 | LXC005609.1 | 17 | 1925658-1926833 | 1176 | 1 | 8.40 | 391 | 43074.6 | 62.57 | -0.474 |
| GlAPC4 | LXC007689.1 | 25 | 544705-546898 | 2194 | 2 | 5.24 | 701 | 76967.2 | 37.77 | -0.256 |
| GlgrrA | LXC006778.1 | 21 | 1528085-1530532 | 2448 | 1 | 4.94 | 815 | 88402.1 | 54.67 | -0.217 |
| GlTME3 | LXC001652.1 | 4 | 401407-403137 | 1731 | 1 | 6.54 | 576 | 64676.1 | 39.24 | 0.176 |
