# Supplementary material for: Identification, characterization and expression profiles of E2 and E3 gene superfamilies during the development of tetrasporophytes in Gracilariopsis lemaneiformis (Rhodophyta)
Source: BMC Genomics. 2023 Sep 18;24:549. doi: 10.1186/s12864-023-09639-0 (PMC10506303; doi:10.1186/s12864-023-09639-0)
Supplement: Supplementary file 9 — Additional file 9: Supplementary Table S3. Genes of ubiquitin mediated proteolysis in cultivars 981 and ZC. [file 12864_2023_9639_MOESM9_ESM.docx]

**Supplementary Table S3.** Genes of ubiquitin mediated proteolysis in cultivars 981 and ZC

| **Gene ID** | **Pathway Gene ID** | **Pathway ID** | | **Pathway name** |  |
| --- | --- | --- | --- | --- | --- |
| LXC002530 | CHC_T00000123001 | ccp04120 | Ubiquitin mediated proteolysis | | |
| LXC005528 | CHC_T00001321001 | ccp04120 | Ubiquitin mediated proteolysis | | |
| LXC000983 | CHC_T00002169001 | ccp04120 | Ubiquitin mediated proteolysis | | |
| LXC005474 | CHC_T00002227001 | ccp04120 | Ubiquitin mediated proteolysis | | |
| LXC000517 | CHC_T00002451001 | ccp04120 | Ubiquitin mediated proteolysis | | |
| LXC005471 | CHC_T00002791001 | ccp04120 | Ubiquitin mediated proteolysis | | |
| LXC001846 | CHC_T00002848001 | ccp04120 | Ubiquitin mediated proteolysis | | |
| LXC004229 | CHC_T00002851001 | ccp04120 | Ubiquitin mediated proteolysis | | |
| LXC007496 | CHC_T00002999001 | ccp04120 | Ubiquitin mediated proteolysis | | |
| LXC007742 | CHC_T00003086001 | ccp04120 | Ubiquitin mediated proteolysis | | |
| LXC003641 | CHC_T00003204001 | ccp04120 | Ubiquitin mediated proteolysis | | |
| LXC007453 | CHC_T00003230001 | ccp04120 | Ubiquitin mediated proteolysis | | |
| LXC003348 | CHC_T00003628001 | ccp04120 | Ubiquitin mediated proteolysis | | |
| NA | CHC_T00003768001 | ccp04120 | Ubiquitin mediated proteolysis | | |
| LXC007310 | CHC_T00003849001 | ccp04120 | Ubiquitin mediated proteolysis | | |
| LXC004082 | CHC_T00004000001 | ccp04120 | Ubiquitin mediated proteolysis | | |
| LXC001902 | CHC_T00004183001 | ccp04120 | Ubiquitin mediated proteolysis | | |
| LXC002797 | CHC_T00004368001 | ccp04120 | Ubiquitin mediated proteolysis | | |
| NA | CHC_T00004379001 | ccp04120 | Ubiquitin mediated proteolysis | | |
| LXC006217 | CHC_T00004619001 | ccp04120 | Ubiquitin mediated proteolysis | | |
| NA | CHC_T00004841001 | ccp04120 | Ubiquitin mediated proteolysis | | |
| LXC006224 | CHC_T00004956001 | ccp04120 | Ubiquitin mediated proteolysis | | |
| LXC006388 | CHC_T00005234001 | ccp04120 | Ubiquitin mediated proteolysis | | |
| LXC002777 | CHC_T00005501001 | ccp04120 | Ubiquitin mediated proteolysis | | |
| LXC003528 | CHC_T00005520001 | ccp04120 | Ubiquitin mediated proteolysis | | |
| LXC003664 | CHC_T00005531001 | ccp04120 | Ubiquitin mediated proteolysis | | |
| NA | CHC_T00005632001 | ccp04120 | Ubiquitin mediated proteolysis | | |
| LXC002718 | CHC_T00005963001 | ccp04120 | Ubiquitin mediated proteolysis | | |
| LXC001238 | CHC_T00006119001 | ccp04120 | Ubiquitin mediated proteolysis | | |
| LXC003945 | CHC_T00006277001 | ccp04120 | Ubiquitin mediated proteolysis | | |
| LXC006784 | CHC_T00006794001 | ccp04120 | Ubiquitin mediated proteolysis | | |
| NA | CHC_T00006960001 | ccp04120 | Ubiquitin mediated proteolysis | | |
| LXC003739 | CHC_T00007109001 | ccp04120 | Ubiquitin mediated proteolysis | | |
| LXC003378 | CHC_T00007378001 | ccp04120 | Ubiquitin mediated proteolysis | | |
| LXC001579 | CHC_T00007642001 | ccp04120 | Ubiquitin mediated proteolysis | | |
| NA | CHC_T00007701001 | ccp04120 | Ubiquitin mediated proteolysis | | |
| LXC003802 | CHC_T00008443001 | ccp04120 | Ubiquitin mediated proteolysis | | |
| LXC000248 | CHC_T00008502001 | ccp04120 | Ubiquitin mediated proteolysis | | |
| LXC003800 | CHC_T00008644001 | ccp04120 | Ubiquitin mediated proteolysis | | |
| LXC001235 | CHC_T00008828001 | ccp04120 | Ubiquitin mediated proteolysis | | |
| LXC003689 | CHC_T00008984001 | ccp04120 | Ubiquitin mediated proteolysis | | |
| NA | CHC_T00009096001 | ccp04120 | Ubiquitin mediated proteolysis | | |
| LXC006810 | CHC_T00009134001 | ccp04120 | Ubiquitin mediated proteolysis | | |
| LXC005243 | CHC_T00009159001 | ccp04120 | Ubiquitin mediated proteolysis | | |
| LXC006232 | CHC_T00009189001 | ccp04120 | Ubiquitin mediated proteolysis | | |
| LXC003223 | CHC_T00009192001 | ccp04120 | Ubiquitin mediated proteolysis | | |
| LXC001188 | CHC_T00009306001 | ccp04120 | Ubiquitin mediated proteolysis | | |
| LXC005281 | CHC_T00009358001 | ccp04120 | Ubiquitin mediated proteolysis | | |
| LXC006581 | CHC_T00009466001 | ccp04120 | Ubiquitin mediated proteolysis | | |
| NA | CHC_T00009526001 | ccp04120 | Ubiquitin mediated proteolysis | | |
| LXC002385 | CHC_T00009552001 | ccp04120 | Ubiquitin mediated proteolysis | | |
| LXC001158 | CHC_T00010007001 | ccp04120 | Ubiquitin mediated proteolysis | | |
| LXC005652 | CHC_T00010295001 | ccp04120 | Ubiquitin mediated proteolysis | | |
| LXC007705 | CHC_T00010346001 | ccp04120 | Ubiquitin mediated proteolysis | | |
| novel.275 | CHC_T00009526001 | ccp04120 | Ubiquitin mediated proteolysis | | |

NA: Not Available
