# Supplementary material for: Identification, characterization and expression profiles of E2 and E3 gene superfamilies during the development of tetrasporophytes in Gracilariopsis lemaneiformis (Rhodophyta)
Source: BMC Genomics. 2023 Sep 18;24:549. doi: 10.1186/s12864-023-09639-0 (PMC10506303; doi:10.1186/s12864-023-09639-0)
Supplement: Supplementary file 10 — Additional file 10: Supplementary Table S4. Genes of ubiquitin mediated proteolysis in strains WLP-1 and WT. [file 12864_2023_9639_MOESM10_ESM.docx]

**Supplementary Table S4** Genes of ubiquitin mediated proteolysis in strains WLP-1 and WT

| **Gene ID** | **Pathway Gene ID** | **Pathway ID** | | **Pathway name** |  |
| --- | --- | --- | --- | --- | --- |
| novel.614 | CHC_T00004207001 | ccp04120 | Ubiquitin mediated proteolysis | | |
| LXC002111 | CHC_T00001321001 | ccp04120 | Ubiquitin mediated proteolysis | | |
| LXC007126 | CHC_T00005963001 | ccp04120 | Ubiquitin mediated proteolysis | | |
| LXC004899 | CHC_T00002851001 | ccp04120 | Ubiquitin mediated proteolysis | | |
| LXC004898 | CHC_T00002851001 | ccp04120 | Ubiquitin mediated proteolysis | | |
| LXC007307 | CHC_T00003086001 | ccp04120 | Ubiquitin mediated proteolysis | | |
| LXC006009 | CHC_T00004956001 | ccp04120 | Ubiquitin mediated proteolysis | | |
| LXC007065 | CHC_T00005501001 | ccp04120 | Ubiquitin mediated proteolysis | | |
| LXC000562 | CHC_T00008828001 | ccp04120 | Ubiquitin mediated proteolysis | | |
| LXC002619 | CHC_T00009358001 | ccp04120 | Ubiquitin mediated proteolysis | | |
| LXC005745 | CHC_T00002851001 | ccp04120 | Ubiquitin mediated proteolysis | | |
| LXC002801 | CHC_T00002451001 | ccp04120 | Ubiquitin mediated proteolysis | | |
| LXC001043 | CHC_T00002169001 | ccp04120 | Ubiquitin mediated proteolysis | | |
| LXC001261 | CHC_T00007378001 | ccp04120 | Ubiquitin mediated proteolysis | | |
| LXC003681 | CHC_T00004183001 | ccp04120 | Ubiquitin mediated proteolysis | | |
| LXC001690 | CHC_T00008828001 | ccp04120 | Ubiquitin mediated proteolysis | | |
| LXC007451 | CHC_T00008984001 | ccp04120 | Ubiquitin mediated proteolysis | | |
| LXC000422 | CHC_T00007701001 | ccp04120 | Ubiquitin mediated proteolysis | | |
| LXC000515 | CHC_T00009306001 | ccp04120 | Ubiquitin mediated proteolysis | | |
| LXC007427 | CHC_T00005531001 | ccp04120 | Ubiquitin mediated proteolysis | | |
| LXC004414 | CHC_T00002851001 | ccp04120 | Ubiquitin mediated proteolysis | | |
| LXC004411 | CHC_T00006794001 | ccp04120 | Ubiquitin mediated proteolysis | | |
| LXC006016 | CHC_T00004619001 | ccp04120 | Ubiquitin mediated proteolysis | | |
| LXC007559 | CHC_T00008644001 | ccp04120 | Ubiquitin mediated proteolysis | | |
| LXC004385 | CHC_T00009134001 | ccp04120 | Ubiquitin mediated proteolysis | | |
| LXC001417 | CHC_T00009192001 | ccp04120 | Ubiquitin mediated proteolysis | | |
| LXC003663 | CHC_T00010349001 | ccp04120 | Ubiquitin mediated proteolysis | | |
| LXC005843 | CHC_T00005234001 | ccp04120 | Ubiquitin mediated proteolysis | | |
| LXC007497 | CHC_T00007109001 | ccp04120 | Ubiquitin mediated proteolysis | | |
| LXC007561 | CHC_T00008443001 | ccp04120 | Ubiquitin mediated proteolysis | | |
| LXC000485 | CHC_T00010007001 | ccp04120 | Ubiquitin mediated proteolysis | | |
| LXC006951 | CHC_T00009466001 | ccp04120 | Ubiquitin mediated proteolysis | | |
| LXC005606 | CHC_T00002791001 | ccp04120 | Ubiquitin mediated proteolysis | | |
| LXC006344 | CHC_T00008502001 | ccp04120 | Ubiquitin mediated proteolysis | | |
| LXC007342 | CHC_T00010346001 | ccp04120 | Ubiquitin mediated proteolysis | | |
| LXC005188 | CHC_T00006277001 | ccp04120 | Ubiquitin mediated proteolysis | | |
| LXC005050 | CHC_T00004000001 | ccp04120 | Ubiquitin mediated proteolysis | | |
| LXC007044 | CHC_T00004368001 | ccp04120 | Ubiquitin mediated proteolysis | | |
| LXC006793 | CHC_T00003230001 | ccp04120 | Ubiquitin mediated proteolysis | | |
| LXC005744 | CHC_T00002851001 | ccp04120 | Ubiquitin mediated proteolysis | | |
| LXC007722 | CHC_T00000123001 | ccp04120 | Ubiquitin mediated proteolysis | | |
| LXC001292 | CHC_T00003628001 | ccp04120 | Ubiquitin mediated proteolysis | | |
| LXC000565 | CHC_T00006119001 | ccp04120 | Ubiquitin mediated proteolysis | | |
| LXC000691 | CHC_T00007642001 | ccp04120 | Ubiquitin mediated proteolysis | | |
| LXC005609 | CHC_T00002227001 | ccp04120 | Ubiquitin mediated proteolysis | | |
| novel.359 | CHC_T00010349001 | ccp04120 | Ubiquitin mediated proteolysis | | |
| novel.601 | CHC_T00004207001 | ccp04120 | Ubiquitin mediated proteolysis | | |
| LXC001982 | CHC_T00010295001 | ccp04120 | Ubiquitin mediated proteolysis | | |
| LXC002581 | CHC_T00009159001 | ccp04120 | Ubiquitin mediated proteolysis | | |
| LXC006683 | CHC_T00003849001 | ccp04120 | Ubiquitin mediated proteolysis | | |
| LXC000176 | CHC_T00002851001 | ccp04120 | Ubiquitin mediated proteolysis | | |

NA: Not Available.
