# Supplementary material for: Identification, characterization and expression profiles of E2 and E3 gene superfamilies during the development of tetrasporophytes in Gracilariopsis lemaneiformis (Rhodophyta)
Source: BMC Genomics. 2023 Sep 18;24:549. doi: 10.1186/s12864-023-09639-0 (PMC10506303; doi:10.1186/s12864-023-09639-0)
Supplement: Supplementary file 11 — Additional file 11: Supplementary Table S5. List of primer sequences for E2 genes DNA and cDNA sequences in Gp. lemaneiformis. [file 12864_2023_9639_MOESM11_ESM.docx]

**Supplementary Table S5** List of primer sequences for E2 genes DNA and cDNA sequences in *Gp. lemaneiformis*

| **Primer name** | | **Sequence** |
| --- | --- | --- |
| LXC003663-F | ACCGACGGAATACGTCTACTTACAA | |
| LXC003663-R | TACACGGCTCCACTGCTACC | |
| LXC006793-F | GATCCCAACCGTGTATCAAAA | |
| LXC006793-R | CCAGCATAGGAATCCAAGAGAA | |
| LXC000128-F | CGCTAACAATGAATTCTCGATCCC | |
| LXC000128-R | TGGCTAAAATTCGGTGCCTTCTG | |
| LXC000565-F | TCGCCCCTTCAAAAAACG | |
| LXC000565-R | CCAGTGTAGATGTGTGTCGGTC | |
| LXC002092-F | CCCCGCCTTCTTTGCTATC | |
| LXC002092-R | CAAAAAAGACAGGGAGCAACG | |
| LXC002619-F | CGGCTGCGCTCCCATCATG | |
| LXC002619-R | CGCAAGCTATGTTTGAACGT | |
| LXC004411-F | AATGAACCCGCACCACAGC | |
| LXC004411-R | CATGGCACGAAGGTTCTACGAC | |
| LXC005843-F | CGCACTTACTCCCTCCCCAT | |
| LXC005843-R | TGAACATCAACGAGAAGACGAC | |
| LXC006951-F | GCGCCCCTCACAAATTCG | |
| LXC006951-R | TGTAAAGCTCGGCTATCACCAA | |
| LXC007342-F | GGAAACATCTACCCACAAAAACTAC | |
| LXC007342-R | ATGAACAGGACGAGGCGAG | |
| LXC007427-F | GCCGTCGCAACTCAACTTC | |
| LXC007427-R | CACAATCTCGTGCTGCATCTG | |
| LXC007561-F | AAGCGCATCATGAAAGTACGTT | |
| LXC007561-R | TAGAGTAGAAGCAGCGGGAGGC | |
| LXC007823-F | ACTATGAAGAGCCAGTTGTTATGTC | |
| LXC007823-R | TCGCAGATGAACGACGC | |
| LXC001783-F | ATGGACGAAGGCACTAAAGACAC | |
| LXC001783-R | TTACTTCTTGAACTTGTCAATTACGCC | |
| LXC006838-F | ATGTCTCAGGTCGCTTCCAAAT | |
| LXC006838-R | TCAAAAATTCCCTAACTCGTATGC | |
