# Supplementary material for: Identification, characterization and expression profiles of E2 and E3 gene superfamilies during the development of tetrasporophytes in Gracilariopsis lemaneiformis (Rhodophyta)
Source: BMC Genomics. 2023 Sep 18;24:549. doi: 10.1186/s12864-023-09639-0 (PMC10506303; doi:10.1186/s12864-023-09639-0)
Supplement: Supplementary file 12 — Additional file 12: Supplementary Table S6. List of primer sequences for E3 genes cDNA sequences in Gp. lemaneiformis. [file 12864_2023_9639_MOESM12_ESM.docx]

**Supplementary Table S6** List of primer sequences for E3 genes cDNA sequences in *Gp. lemaneiformis*

| Primer name | Sequence |
| --- | --- |
| LXC006375.1-F | GGACCAAACGCACAAAAGAA |
| LXC006375.1-R | CCAAGCCGTGCCCCAT |
| LXC007412.2-F  LXC007412.2-R  LXC001417.1-F | ATCAACAGGCGTTCAGAGGTC  CTTCCAAAACCAAAGTGAGTATGTG  TTCTTTAATCGCCTAAACCCC |
| LXC001417.1-R | GCAGATTATGGTGCCCTTGTA |
| LXC000452.1-F | TGGCGTATCATTCAGCTCATC |
| LXC000452.1-R | TTTCCGTCTCAAAAGAACTCG |
| LXC006083.1-F | TCCAACCCGTCTACATCCTACT |
| LXC006083.1-R | CATGGTATCGTCAAGCTGAGTC |
| LXC008086.1-F | CCCGCCCGTCTTTCCTT |
| LXC008086.1-R | TCACAGCTACTTGCGAGTTCTATT |
| LXC000691.2-F | TTGCGTTCCAACATCGTCA |
| LXC000691.2-R | AAAGGGTTAAAGCGCTAGCTT |
| LXC000426.1-F | TCCCACGCTGCCTTCTCT |
| LXC000426.1-R | CTTTCTAGTTCGGACATGGGTTA |
| LXC006806.1-F | CGGGGTTGGAGGGGATT |
| LXC006806.1-R | TGTGTTATGGGATGAAAGACGG |
| LXC001261.1-F | AGATTTACCCCCTAACCTTGG |
| LXC001261.1-R | ACCTCCGTTCGTATGTCCTC |
| LXC006812.1-F | AGGCAGCAGCGGTTTGTC |
| LXC006812.1-R | CCTTCTTTGAGACTTTTGTTCCAC |
| LXC007042.1-F | GCTGTAGCTTTCCTACCAACGA |
| LXC007042.1-R | GGAGAACTGGCAAGTGATGATTTA |
| LXC000754.1-F | TGTCCTTAACTGATGTTGGCACT |
| LXC000754.1-R | GGGAGCTGAAACGATTTACCTG |
| LXC000422.1-F | TTCGCTAACCTTCGCTCCCAC |
| LXC000422.1-R | GTGTGCGGTCTGACAATGCGG |
| LXC000486.1-F | CCAATCAAATGTCAACCAACGA |
| LXC000486.1-R | CCAATCAAATGTCAACCAACGA |
| LXC005609.1-F | CTGCTTCGTCCTAGATACCAAGAGT |
| LXC005609.1-R | CGCCTGAACCTGCAAATGAATA |
| LXC007689.1-F | CGTCAAGGTACTGCGTCGTCC |
| LXC007689.1-R | CCCATGCCTCATTCCAAACC |
| LXC006778.1-F | GATGATAGTGAGGTTTGTGAGGC |
| LXC006778.1-R | CATCTCTCTGCTTTCTTTCTCTACA |
| LXC005534.3-F | CAACCCGCCCCAAAAAA |
| LXC005534.3-R | CCGATAACATCAGCAGGTCAGA |
| LXC003681.1-F | AGTTACATTGAGAAGAACACCCC |
| LXC003681.1-R | CGTTGTCCCAAAGAACATCC |
| LXC003991.1-F | ATGTAGAGAGGAGTTCTTTGGTAGG |
| LXC003991.1-R | AAGGCGATGTGAAGGCGT |
| LXC001490.1-F | ACCTCTCAAGCCTTCCCGT |
| LXC001490.1-R | CGTCGAAAGCATCAGATAACC |
| LXC006344.1-F | CGCTAGCACTGGGAGATTTC |
| LXC006344.1-R | ACTTCCTTCTTCAACGTACTTTCGC |
| LXC001726.1-F | TTGCTTCCTGCTCCTCCTT |
| LXC001726.1-R | TCACAGCCTTTCTGCACTCA |
| LXC004838.1-F | ACCAGAGAAGCGTTAGCGGC |
| LXC004838.1-R | ACGGGATGTGGGTTCAAGAC |
| LXC004472.1-F | CAAACCAGTGCGAACACAAGC |
| LXC004472.1-R | CGCCACAATTTTTCATTCTCTCTA |
| LXC001227.1-F | AGCTTCGTTTCACGCTGTCC |
| LXC001227.1-R | TCCAGGTTTCGTGCTACAAGTG |
| LXC006806.2-F | CAGCACCATTCACCACCAGAT |
| LXC006806.2-R | TTGACCTCCCCGTCCGTTT |
| LXC000111.1-F | GGCATCATCCAGCATAAACGC |
| LXC000111.1-R | ACCTACATCGCAACCCCAGC |
| LXC008198.1-F | TTCCAAACTTTGCGCGTCC |
| LXC008198.1-R | AACAAAAGCCCCCTGTAACCA |
| LXC001519.1-F | AGGACGCCTTGTGCCGA |
| LXC001519.1-R | CCCTCTCCCCCACCTAACTCT |
| LXC000515.1-F | GCTATCAATGCTGAGTTGGACA |
| LXC000515.1-R | GTCCAACTCAGCATTGATAGCA |
| LXC007451.1-F | CTCTTCCAACCGATGACCTTT |
| LXC007451.1-R | TCTACCTCTGTCCAACACCCAC |
| LXC006054.1-F | ATTTCGCTGGCACGCAC |
| LXC006054.1-R | CCTGGACATCCCAACACAAG |
| LXC007039.1-F | CAGCGGTTAGCGAAGAAAGC |
| LXC007039.1-R | CTCCAAACAAACAGCACAAACATCA |
| LXC001652.1-F | GTCCACCCTCGACACGGTAA |
| LXC001652.1-R | AGATGTCCAAGGCACAGGTAAGA |
| LXC000861.1-F | GTAACTGAATATGGGGAGCTTATGTG |
| LXC000861.1-R | CGCTGCTGTGTATGTATGATGGT |
| LXC006211.1-F | GACCCTTCAGCCGAACACAA |
| LXC006211.1-R | TGAGACAGGATGGCGACAAAT |
| LXC005534.1-F | CTGGCAGGACTGAAAAGGGTG |
| LXC005534.1-R | GCAAGGCGGTGCGTAACAA |
| LXC005534.2-F | CTCTGGCAGGACTGAAAAGGG |
| LXC005534.2-R | ATCGTCTGGGGCACATCTCG |
| LXC002035.1-F | TCCGATGAGAAGCAGTTAGCCA |
| LXC002035.1-R | TCCTTTTCGTAGGTTTCCAGACA |
| LXC002549.1-F | CAACCACTGCTTCCACCAAAA |
| LXC002549.1-R | ACCATTCCAGCCAGCTCTGA |
| LXC007722.1-F | ATGGAGGACTTTGACAGCAATACA |
| LXC007722.1-R | TTTTCAGAGCCAAGAAACCTACACT |
| LXC002149.1-F | ACATGCCTCGTCTTACATCTGGA |
| LXC002149.1-R | CGCACATTGTTTGGGGGTTAT |
| LXC005020.1-F | GCCGTAGATGACAGCATTTGG |
| LXC005020.1-R | AGGTTGCCTTCTCCTTCGTTC |
| LXC000912.1-F | AGCAAAGCCGTCAGAAGTCG |
| LXC000912.1-R | AACCGCTGTTTACTGCCCAC |
| LXC007412.1-F | CGGGAAGTGAACGTCAAAAA |
| LXC007412.1-R | CTGAGGCGGTAGTAGTGTGGATA |
| LXC000380.1-F | CTGTGACCGCTGCGAGTTT |
| LXC000380.1-R | CCATGAAGACCTTGTAGGTAATGAA |
