# Supplementary material for: Identification, characterization and expression profiles of E2 and E3 gene superfamilies during the development of tetrasporophytes in Gracilariopsis lemaneiformis (Rhodophyta)
Source: BMC Genomics. 2023 Sep 18;24:549. doi: 10.1186/s12864-023-09639-0 (PMC10506303; doi:10.1186/s12864-023-09639-0)
Supplement: Supplementary file 13 — Additional file 13: Supplementary Table S7. List of primer sequences for qPCR of E2 genes in Gp. lemaneiformis. [file 12864_2023_9639_MOESM13_ESM.docx]

**Supplementary Table S7** List of primer sequences for qPCR of E2 genes in *Gp. lemaneiformis*

| **Primer name** | **Sequence** |
| --- | --- |
| LXC001783-F | CGGTGACCTATCCTGAAACGG |
| LXC001783-R | CAATGCCAAAGCGAGGGAC |
| LXC002092-F | GCAAGGCAGAGGAGTGGACAA |
| LXC002092-R | CGGATGGATGCGATTCGTTTT |
| LXC004411-F | ATCCTATGGAAGCCCCCGA |
| LXC004411-R | TGCTGACGATGCTGAGACAAACT |
| LXC006838-F | CAGAAAACGAAGTATCGGGGG |
| LXC006838-R | GTAGGCTTTGCTGGTCGTGAGA |
| LXC007342-F | CATCTGGTATCAGCGGAGCG |
| LXC007342-R | GGCGGTTTGTTGGGGTAGTC |
| LXC007427-F | GTCCTCCGCATCCTCATCCA |
| LXC007427-R | GGCGTTATCGGGTCTCGTTACT |
| LXC007561-F | ATTCGCACCGTCTTGCTCTCA |
| LXC007561-R | CGTATTGCTTGGTCCAGTCTCG |
