# Supplementary material for: Identification, characterization and expression profiles of E2 and E3 gene superfamilies during the development of tetrasporophytes in Gracilariopsis lemaneiformis (Rhodophyta)
Source: BMC Genomics. 2023 Sep 18;24:549. doi: 10.1186/s12864-023-09639-0 (PMC10506303; doi:10.1186/s12864-023-09639-0)
Supplement: Supplementary file 14 — Additional file 14: Supplementary Table S8. List of primer sequences for qPCR of E3 genes in Gp. lemaneiformis. [file 12864_2023_9639_MOESM14_ESM.docx]

**Supplementary Table S8** List of primer sequences for qPCR of E3 genes in *Gp. lemaneiformis*

| **Primer name** | | **Sequence** |
| --- | --- | --- |
| 515-s | GCGGACAGGTCGAAAGGATG | |
| 515-a | GGAAGAACTGAGGCTGTTGCTG | |
| 853-s | GCGACGATCTTCTAGGTCCATT | |
| 853-a | CGCTGTTGCCTTTCTTTATCACTT | |
| 912-s | GAGCAAAGCCGTCAGAAGTCG | |
| 912-a | CACCTGCACCCTCCCGTTTA | |
| 1261-s | CATAACGTCCGAAATACTACAGCG | |
| 1261-a | TCCGTCACCAGTCATTCCACTAA | |
| 1490-s | ACTTGACCGCTATGCTGAACG | |
| 1490-a | CATTGGTGTCATCGCCATTTT | |
| 1652-s | CTGCTGCTCCCGCTCCTAC | |
| 1652-a | CGAATCCGGTCGATTTATGG | |
| 1726-s | AACAATCCCAAGGCTCCCG | |
| 1726-a | TGAGCATCGCCAACACCC | |
| 2549-s | CAACCACTGCTTCCACCAAAA | |
| 2549-a | ACCATTCCAGCCAGCTCTGA | |
| 3681-s | CGAGTGAGGGAGGGTAGTTTTG | |
| 3681-a | ATCCGTGATGCCATCGACTTA | |
| 4472-s | TACATAAGGGTCTATCCAAACAGGG | |
| 4472-a | CGAACTAAACCACCGAAAGCAA | |
| 5534-s | CGAGATGTGCCCCAGACGAT | |
| 5534-a | GCAAGGCGGTGCGTAACAA | |
| 6054-s | GCTCCGTCTTCGAGGTTTACAT | |
| 6054-a | CGCCATTTCCTGGTTTTGC | |
| 6778-s | GTTCGCCACGTCACCATCA | |
| 6778-a | GGCAAAGCATTCGGTCAAGG | |
| 6806-s | CCGAACGGAGGAGATTGGA | |
| 6806-a | CGGAACTCTGAACTTGGTAGGC | |
| 7039-s | CAGCGGTTAGCGAAGAAAGC | |
| 7039-a | CTCCAAACAAACAGCACAAACA | |
| 7412-s | CGTGGCGATGCGGTCTTT | |
| 7412-a | TGCGTTGCTTCTGTAATCTTTAGG | |
| 8086-s | GAGGAAGCGGGTATTGATGGT | |
| 8086-a | CAAGGAACGCAAACTGGGAC | |
